# Insights into the transglucosylation activity of α-glucosidase from *Schwanniomyces occidentalis*

**DOI:** 10.1007/s00253-024-13262-8

**Published:** 2024-08-17

**Authors:** Zoran Merdzo, Egle Narmontaite, Jose L. Gonzalez-Alfonso, Ana Poveda, Jesus Jimenez-Barbero, Francisco J. Plou, María Fernández-Lobato

**Affiliations:** 1 Centro de Biología Molecular Severo Ochoa, Departamento de Biología Molecular (UAM-CSIC), Universidad Autónoma de Madrid, C/ Nicolás Cabrera, 1. Campus Cantoblanco, 28049 Madrid, Spain; 2 Instituto de Catálisis y Petroleoquímica (CSIC), C/ Marie Curie, 2., 28049 Madrid, Spain; 3 CIC bioGUNE, Basque Research and Technology Alliance (BRTA), 48160 Derio, Spain; 4 Ikerbasque. Basque Foundation for Science, 48009 Bilbao, Spain

**Keywords:** α-Glucosidase, *Schwanniomyces occidentalis*, Transglycosylation, Hetero-glucooligosaccharides, Piceid

## Abstract

**Abstract:**

The α-glucosidase from *Schwanniomyces occidentalis* (GAM1p) was expressed in *Komagataella phaffii* to about 70 mg/L, and its transferase activity studied in detail. Several isomaltooligosaccharides (IMOS) were formed using 200 g/L maltose. The major production of IMOS (81.3 g/L) was obtained when 98% maltose was hydrolysed, of which 34.8 g/L corresponded to isomaltose, 26.9 g/L to isomaltotriose, and 19.6 g/L to panose. The addition of glucose shifted the IMOS synthesis towards products containing exclusively α(1 → 6)-linkages, increasing the production of isomaltose and isomaltotriose about 2–4 fold, enabling the formation of isomaltotetraose, and inhibiting that of panose to about 12 times. In addition, the potential of this enzyme to glycosylate 12 possible hydroxylated acceptors, including eight sugars and four phenolic compounds, was evaluated. Among them, only sucrose, xylose, and piceid (a monoglucosylated derivative of resveratrol) were glucosylated, and the main synthesised products were purified and characterised by MS and NMR. Theanderose, α(1 → 4)-D-glucosyl-xylose, and a mixture of piceid mono- and diglucoside were obtained with sucrose, xylose, and piceid as acceptors, respectively. Maximum production of theanderose reached 81.7 g/L and that of the glucosyl-xylose 26.5 g/L, whereas 3.4 g/L and only 1 g/L were produced of the piceid mono- and diglucoside respectively.

**Key points:**

*• Overexpression of a yeast α-glucosidase producing novel molecules.*

*• Yeast enzyme producing the heterooligosaccharides theanderose and glucosyl-xylose.*

*• Glycosylation of the polyphenol piceid by a yeast α-glucosidase.*

**Supplementary Information:**

The online version contains supplementary material available at 10.1007/s00253-024-13262-8.

## Introduction

The functional food and nutraceutical markets are tremendously growing because of the increasing social demand for products that possess a beneficial impact on health (Barros et al. 2022; Ji et al. 2022; Shinde and Vamkudoth 2021). Bioactive oligosaccharides such as fructooligosaccharides, xylooligosaccharides, galactooligosaccharides, and isomaltooligosaccharides (among others) are produced commercially through natural source extraction and/or enzymatic synthesis (Meyer et al. 2015). Isomaltooligosaccharides (IMOS) are glucosyl-saccharides formed by units of D-glucose linked by α(1 → 6), α(1 → 2), α(1 → 3), and α(1 → 4) glucosidic bonds forming branched or even cyclic oligosaccharides with a degree of polymerisation of 2–10 (Sorndech et al. 2018). IMOS have shown several health benefits associated with their prebiotic properties, including low glycemic index, improved bowel function, bifidogenic response, immunomodulatory effect, cholesterol regulation, and mineral absorption (Chen et al. 2022; Gourineni et al. 2018; Liu et al. 2019; Palaniappan and Emmambux 2021; Villéger et al. 2022). One of the most common methods of IMOS production in the industry is the transglycosylation of saccharified starch with an α-glucosidase usually from *Aspergillus* sp. (Kwon et al. 2011).

Glycoside hydrolases (GH) from the structural families 13, 31, 57, 66, and 70 (www.cazy.org) can produce different types of IMOS using sucrose, maltose, maltooligosaccharides, starch, or dextran as substrate (Casa-Villegas et al. 2018). The α-glucosidases (E.C. 3.2.1.20) belong to the families GH13 or GH31 and are characterised by a (β/α)_8_-barrel structure (Svensson and Janecek 2020; Zhang and Williams 2020). These enzymes hydrolyse oligosaccharides linked by α(1 → 4) glycosidic bonds from its non-reducing termini releasing glucose units, and in saturating substrate conditions can also display transglycosylating activity forming different types of IMOS with potential prebiotic properties (Kawano et al. 2020; Zhou et al. 2009). Some α-glucosidase can also glycosylate other carbohydrates, which act as acceptors yielding new oligosaccharides (Alatorre-Santamaría et al. 2019; Nimpiboon et al. 2011). Moreover, they can even utilise vitamins and phenolic compounds as glucosyl-acceptors, improving their physicochemical properties (Chen et al. 2019; Qi et al. 2021). Commercially available IMOS are usually composed of a very varying mixture of oligosaccharides with distinct degree of polymerisation showing a variable digestibility and discrepant health benefits (Madsen et al. 2017). Digestibility of IMOS depends more on the linkage type connecting the monosaccharide units than the degree of polymerisation (Hu et al. 2020). Consequently, improved specific formulation and optimisation of the IMOS synthesis is needed to meet the increasing demand of these molecule types.

The α-glucosidase GAM1p from *Schwanniomyces occidentalis* ATCC26074, a GH31 protein of 960 amino acids, was previously poorly expressed in *Pichia pastoris* (also *Komagataella phaffii*) to about 0.2 U/mL and kinetically analysed using small substrates such as maltose and isomaltose, as well as soluble starch (Sato et al. 2005). The transferase activity of this enzyme was also proved using maltose as substrate, with maltotriose (Glc-α(1 → 4)-Glc-α(1 → 4)-Glc), isomaltose (Glc-α(1 → 6)-Glc), and panose (Glc-α(1 → 6)-Glc-α(1 → 4)-Glc) as the main transglycosylation products (Song et al. 2013), which were identified but not quantified. In this study, the capacity of GAM1p to produce IMOS from maltose was analysed in detail as well as its ability to glycosylate different saccharide acceptors and phenolic compounds. The new products obtained by glycosylation of sucrose and xylose, as well as piceid (potent antioxidant related to resveratrol), were purified and characterised by MS and NMR, data that significantly increase the biotechnological potential of GAM1p, and that make it a promising tool in obtaining new glycosylated compounds.

## Material and methods

### Microorganisms, culture media, and growth conditions

*S. occidentalis* ATCC 26077 was maintained and cultured on yeast extract peptone dextrose (YEPD) solid medium (1% yeast extract, 1% peptone, 2% glucose, 2% agar; all w/v). *K. phaffii* GS115 (*his4*^*−*^; also *P. pastoris* GS115; Invitrogen, Carlsbad, CA, USA) was used as protein expression host and was initially cultured in YEPD liquid medium (1% yeast extract, 1% peptone, 2% glucose; all w/v) at 30 °C. Transformants of *K. phaffii* were selected on minimal dextrose (MD) medium (1.34% yeast nitrogen bases w/o amino acids, 4 × 10^*−*5^% biotin, 2% glucose; all w/v). Expression of the GAM1p protein was induced in buffered minimal methanol (BMM) medium after growing transformants in buffered minimal glycerol (BMG) medium (both media same composition as MD but in potassium phosphate 100 mM pH 6.0 and 0.5% methanol or 1% glycerol, respectively). Growth was monitored spectrophotometrically at a wavelength of 600 nm (OD_600_). *Escherichia coli* DH5α (Invitrogen, Carlsbad, CA, USA) was used as cloning host for DNA manipulations using standard techniques.

### DNA manipulation and cloning

The α-glucosidase *GAM1* gene from *S. occidentalis* comprised of 2880 bp (2883 bp with the stop codon) codes a protein of 960 amino acids (UniProt, Q401B1 but Ser845 substituted by Ala) including a signal peptide of 22 residues (calculated using the ExPASy Compute Mw/pI tool). Genomic DNA from *S. occidentalis* was obtained using standard techniques as described previously (Gimeno-Pérez et al. 2021). To express the protein GAM1p in *K. phaffii*, the pIB4 (*HIS4*) vector including the methanol-regulated alcohol oxidase promoter (*AOX1*p) was used as previously referred (Gimeno-Pérez et al. 2015). The gene *GAM1* was amplified using primers GAM1.Fw: 5′-gtgtgtgaattcatgatttttctgaagctgat-3′ and GAM1.Rv: 5′-gtgtgtggatccttaccaagtaatggtgaaa-3′ (*EcoR*I and *Bam*HI restriction sites underlined). Phusion high-fidelity DNA polymerase (NEB, Ipswich, UK) was used for PCR amplification with the following conditions: initial denaturing at 98 °C for 30 s, 10 cycles of 98 °C for 10 s denaturing, 55 °C for 20 s annealing and 72 °C for 1 min 30 s extension, and then another 25 cycles but increasing the annealing temperature to 68 °C and with a final extension at 72 °C for 7 min. Both the PCR product (2919 bp) and the vector pIB4 were digested with *Eco*RI and *Bam*HI (from NEB, Ipswich, UK), and purified using PCR Clean-Up System and agarose gel followed by Wizard SV Gel (Promega, Madison, USA), respectively. The purified digested products were ligated using T4 DNA ligase (NEB, Ipswich, UK) and then, transformed into *E. coli* cells. Colonies including the generated GAM1-pIB4 construction were detected by PCR using the primers 5′AOX: 5′-gactggttccaattgacaagc-3′ and 3′AOX: 5′-gcaaatggcattctgacatcc-3′, both from Sigma-Aldrich (St. Louis, MO, USA), and directed to sequences in the vector flanking the site of insertion, which generates a 3107-bp amplification product. The integrity of the final construction was verified by DNA sequencing (Macrogen, Madrid, Spain).

The GAM1-pIB4 construction was linearised with *Stu*I and transformed into *K. phaffii* by electroporation following the protocol of the manual for protein expression in *Pichia* (Invitrogen, Carlsbad, CA, USA). The integration of the gene *GAM1* in the genome of the yeast transformants was confirmed by PCR using the previously referred 5′AOX and 3′AOX primers. *K. phaffii* transformed with the empty pIB4 vector was also obtained and used as control.

### Protein expression, quantification, and deglycosylation analyses

Expression of GAM1p in *K. phaffii* was analysed using BMM medium and the heterologous protein activity was evaluated by measuring the α-glucosidase activity in the yeast culture filtrates. Initially, transformants carrying the construction GAM1-pIB4 were cultivated at 30 °C in 25 mL of BMG during 24 h, and then escalated to 200 mL in BMM using 1-L flasks. The transformant growth (units at OD_600_; ODU) was evaluated spectrophotometrically. Cells were removed by centrifuging at 1600 × *g* for 15 min, and the extracellular fraction was filtrated with nitrocellulose membrane MF-Millipore 0.45 μm (Millipore Ltd., Ontario, Canada) and then concentrated and fractionated through 50,000 MWCO PES membrane using a Vivaflow 50 system (Sartorius, Göttingen, Germany). When required, proteins were concentrated using Amicon Ultra-Ultracel (50000 MWCO) filters at 5000 × *g* for 20 min (Sorvall SPX 6000 rotor). Proteins were analysed in sodium dodecyl sulphate polyacrylamide gel electrophoresis (SDS-PAGE) 8% (w/v), with ProtoBlue Colloidal Coomassie (National Scientific, Atlanta, GA, USA) staining. Weight markers: Precision Plus Protein Standards Unstained 10–250 kDa (Bio-Rad, Hercules, CA, USA) were used. For the extraction of soluble proteins associated to the cellular fraction, YeastBuster Protein Extraction Reagent (EMD Millipore Corp. Merck KGaA, Darmstadt, Germany) was used using the manufacturer’s conditions. Protein concentration was determined in a NanoDrop 1000 Spectrophotometer (V3.8 Thermo Fisher Scientific Inc., Wilmington, USA) at 280 nm using bovine serum albumin as standard.

For the protein deglycosylation analysis, PNGase F and α(1 → 2,3,6) mannosidase were used according to the manufacturer recommendations (NEB, Massachusetts, USA), for 2 h each in native conditions.

### α-Glucosidase hydrolytic activity assays

Unless otherwise indicated, α-glucosidase activity was determined by detection of glucose produced by maltose hydrolysis. Reactions (50 μL) containing 2 mM maltose in 50 mM sodium acetate (pH 4.5) and 5 μL of enzymatic solution (diluted when necessary to fit into the calibration curve) were incubated at 37 °C for 10 min in 96-well microplates (Greiner Bio-One North America Inc., Monroe, NC, USA) using a VorTemp™ 56 shaking incubator (Labnet International, Edison, NJ, USA) at 400 rpm. After inactivation at 80 °C for 15 min, 200 μL of GOD-POD mix (NZYTech, Lda. Genes and Enzymes, Lisboa, Portugal) was added and reactions were incubated at 50 °C for 20 min. The absorbance was measured at 515 nm in an iMark™ microplate reader (Bio-Rad Laboratories, Hercules, CA, USA). A calibration curve with glucose (0–20 μg/mL) was used. The reactions were always done in triplicate and standard error values of less than 5% were obtained. One unit of α-glucosidase activity (U) was defined as that corresponding to the release of 1 μmol of glucose per minute.

### Transferase activity assays

Transglycosylation reactions (1.5 mL) were carried out using 200 g/L maltose in 50 mM sodium acetate (pH 4.5) and 25 U/mL of GAM1p. To analyse glycosylation of the potential acceptors, 150 and 300 g/L of fructose (or glucosamine, glucose, galactose, lactose, cellobiose, sucrose, and xylose) were used as well as 150 g/L and 200 g/L of maltose as glucosyl donor (in the case of glucose, only 150 g/L of maltose was used) and 25 U/mL of GAM1p. The potential of GAM1p for the glycosylation of phenolic compounds, such as epigallocatechin gallate (EGCG), hydroquinone, hydroxytyrosol, and piceid, was evaluated in reactions containing 25 g/L of the possible acceptor and 150 g/L maltose, except in the case of piceid, which was 7.6 g/L (20 mM) and 300 g/L maltose. In addition, the piceid reaction mixture contained 20% (v/v) DMSO. In these reactions, 50 U/mL of GAM1p was used.

The reaction mixtures were incubated at 37 °C with agitation at 400 rpm in the orbital incubator mentioned above. Aliquots of 0.2 mL were extracted at different times, inactivated at 100 °C for 7 min, and diluted with Milli-Q water or with methanol to reach suitable concentrations for their analysis by HPLC.

### HPLC analysis and purification of the new glucosylated products

All samples were filtrated through 0.45-μm pore nylon-membrane syringe filters (Scharlau, S.L., Sentmenat, Barcelona, Spain). For the analysis of IMOS and heterooligosaccharides, HPLC with a quaternary pump (Delta 600, Waters, Cerdanyola del Vallès, Barcelona, Spain) coupled to a Luna 5-μm 100-Å column (250 mm × 4.6 mm; Phenomenex, Alcobendas, Madrid, Spain) was used. The mobile phase in a linear gradient flow of 1 mL/min was acetonitrile:H_2_O 85:15 (v/v) for 10 min, then acetonitrile:H_2_O 70:30 (v/v) for 25 min with a final 5 min to return to the initial conditions. Detection was performed using an evaporative light-scattering detector (ELSD mod. 1000, Polymer Laboratories, Ltd., Church Stretton, UK) equilibrated at 90 °C. The data obtained was analysed using Millenium 32 and Empower software (v. 1.0, both from Waters Corp.). For the analysis of glycosylated phenolic compounds, a Zorbax Eclipse Plus C18 3.5 μm (Agilent Technologies, Santa Clara, CA, USA) column was employed. The mobile phase was a gradient with acidified acetonitrile containing formic acid and water at 0.1% (v/v) degassed with helium. The gradient started with 5% (v/v) of acetonitrile and reached 40% (v/v) in 12 min. Then, it is maintained at 40% (v/v) for 1 min more and, finally, the gradient came back to the starting conditions during 5 min to re-equilibrate the column for the next injection. The detector was a photodiode array (PDA Varian ProStar 420). The quantification of the phenolic compounds was carried out at 308 nm and the data collected using the Varian Star LC workstation 6.41 software. In both cases, identification and quantification of the different compounds were carried out employing both synthesised or the commercially available standards: glucose, maltose, sucrose, xylose, isomaltose, maltotriose, isomaltotriose (all from Sigma-Aldrich, St Lois, MO, USA), and panose (TCI Europe, Zwijndrecht, Amberes, Belgium). The HPLC analyses were done in duplicate, and each quantification point represents the average of two measurements with standard error ≤ 5%.

New products formed by glucosylation of the acceptors sucrose and xylose were purified by semi-preparative HPLC using a Kromasil-NH2 column (250 × 10 mm, 5 µm) from Análisis Vínicos S.L. (Tomelloso, Spain). Three-way flow splitter (Accurate, LC Packings) was used coupled with ELSD and an acetonitrile:H_2_O (75:25) flow of 4.7 mL/min for 5 min and gradually changed to 70:30 (v/v) and maintained for 40 min, another 5 min with 75:25 was kept until the end of the analysis. The mobile phase of the collected transglycosylation products was evaporated in a R-210 rotavapor (Buchi, Ibérica, Barcelona, Spain) at 65 °C.

Semi-preparative HPLC for piceid glucosides purification was performed using a quaternary pump (model 600, Waters) coupled to an autosampler (Varian ProStar, model 420). The injection volume was 100 µL. The column was a Zorbax Eclipse XDB C-18 column (9.4 × 250 mm, 5 µm, Agilent Technologies) at 40 °C. The column was coupled to a three-way flow splitter (Accurate, LC Packings). The detection of peaks was carried out using a photodiode array detector (ProStar, Varian) at 308 nm. The mobile phase was acetonitrile:H_2_O degassed with helium and acidified with a 0.1% (v/v) of formic acid. The gradient was from a 10% (v/v) of acetonitrile to a 25% (v/v) in 12 min, followed by 8 min to equilibrate the column al the initial conditions. The flow rate was 6.5 mL/min. The solvents of the different fractions were evaporated in a R-210 rotavapor as above.

Purity of the new glycosylated products obtained in this work (from sucrose, xylose and piceid) was assessed by HPLC and mass spectrometry, and in all cases, it was above 95%.

### Mass spectrometry (MS) and nuclear magnetic resonance (NMR)

The purity and molecular weight of the new synthesised molecules were analysed by mass spectroscopy (MS) using MS–ESI with a hybrid quadrupole time of fly (QTOF) analyser (model QSTAR Pulsar I, AB Sciex). Samples were electrospray ionised in positive mode by direct infusion with methanol containing 1% of sodium iodide.

Structure of the purified synthesised sugars was determined by NMR using a combination of standard 1D (^1^H, 1D selective TOCSY, NOESY, and/or ROESY) and 2D (COSY, TOCSY, HSQC, HSQC-TOCSY, HMBC, NOESY/ROESY) experiments. NMR spectra were recorded on a Bruker AV-III 600 spectrometer equipped with a PA-TXI probe with gradients in the *X*,*Y*,*Z*-axis, at a temperature of 298–308 K. The derivatives of sucrose (10.8 mg) and xylose (14 mg) were dissolved in 0.6 mL of D_2_O (ca. 35–70 mM). Chemical shifts were expressed in parts per million (ppm) relative to the TSP-d4 signal used as an internal reference. The piceid glycosylated purified samples (0.5, 1.0, 1.5 mg) were dissolved in 0.6 mL of MeOD-d4 and the residual solvent signal (d 3.31 ppm) was used as an internal reference. Standard Bruker pulse sequences were used. For the HSQC, HSQC-TOCSY, and HMBC experiments, spectra widths were between 5 and 10 ppm for the ^1^H dimension (1–2 K points were used), and 50–110 ppm (256–512 points) for the ^13^C dimension. For the COSY and NOESY homonuclear experiments, the spectral widths were between 4 and 10 ppm, with 1–2 K for F2 and 256–512 points for F1. The mixing time for the NOESY experiment was set to 500 ms, for ROESY, to 300 and 400 ms, and for the TOCSY or HSQC-TOCSY, 80 ms.

## Results

### Heterologous production of the protein GAM1p

**Fig. 1:**
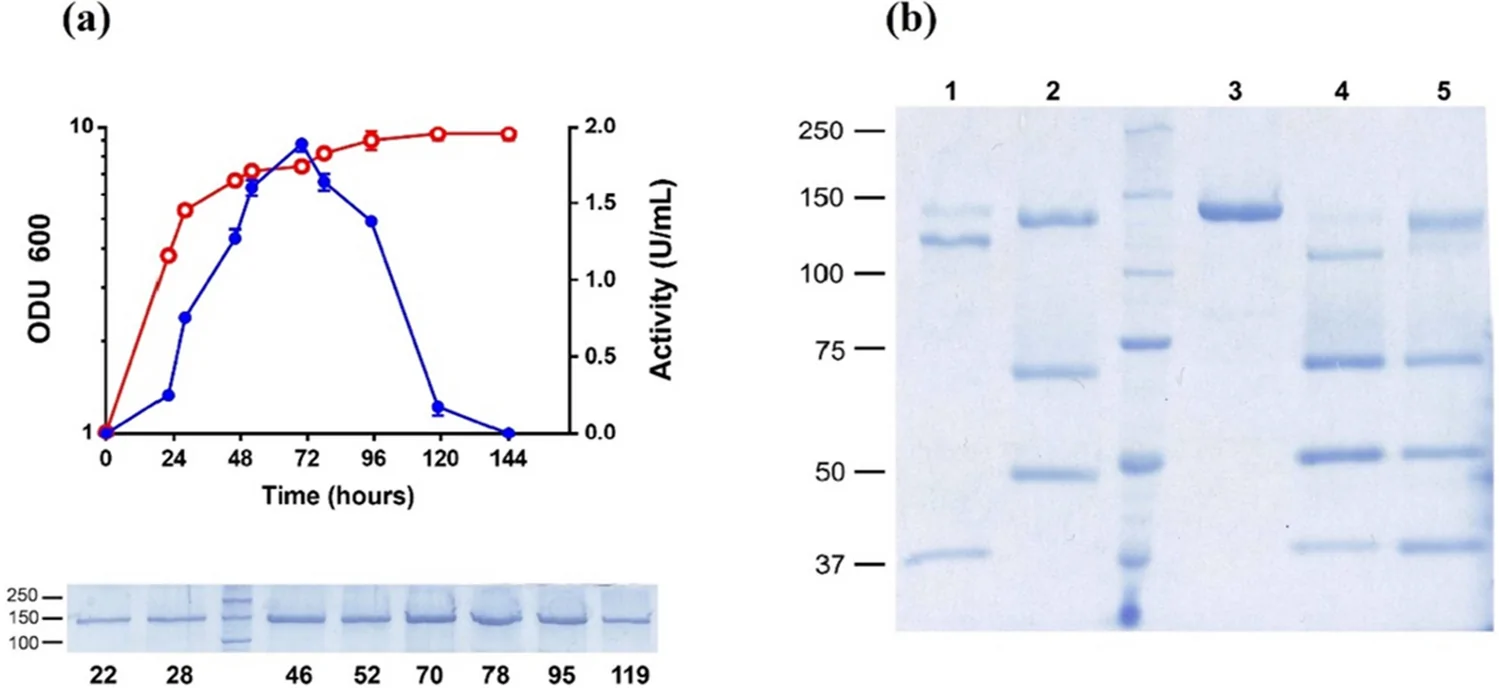
GAM1p heterologous expression and deglycosylation treatment. **a** Time-course of the α-glucosidase produced in *K. phaffii* including construction GAM1-pIB4. Yeast was cultivated in BMM (empty red circles), and extracellular activity (full blue circles) was analysed at the indicated times using maltose as substrate. Each point represents the average of three independent measurements, standard error ≤ 5%. Analysis on SDS-PAGE of the about 143 kDa protein detected at the indicated times of culture (bottom image). **b** Analysis on SDS-PAGE of the heterologous protein purified from the 70-h culture extracellular medium using 50 kDa MWCO membranes (lane 3), treated with PNGase F (500U; 36 kDa; lane 1) and α(1–2,3,6) mannosidase (2U; 44 and 66 kDa; lane 2). Sequential treatment of lane 3 with PNGase F (2 h) and α(1 → 2,3,6) mannosidase (lane 4) and the opposite order (lane 5). Numbers on the left indicate the positions of molecular mass standards (lane between time 28 and 46 h in bottom image of **a** and between lanes 2 and 3 in **b**) in kDa

The α-glucosidase GAM1p from *S. occidentalis* was expressed in *K. phaffii* using a pIB4 derivative vector where the gene *GAM1* was preceded by the *AOX*1 promoter and thus its production was directly controlled by methanol. The α-glucosidase activity of the yeast transformants grown in a methanol-supplemented medium was monitored for 144 h. This activity was already clearly detected in the yeast culture filtrates after 22 h (0.3 U/mL) and its maximum level (1.9 U/mL) was reached after 70 h (Fig. 1a). No α-glucosidase activity associated with the cellular-soluble fraction was detected at any of the times analysed (data not shown). A majority protein band of about 143 kDa was also detected in SDS-PAGE, which increased in intensity with the culture growth and decreased after about 120 h (Fig. 1a and Fig. S1). The maximum amount of the protein GAM1p produced reached 70 mg/L of culture (70–78 h of culture; 27 U/mg) and was apparently purified after a simple concentration step of the extracellular medium through 50-kDa MWCO membranes (Fig. 1b, lane 3).

The theoretical molecular weight of the GAM1p, about 104 kDa (without its export sequence), was clearly lower than that of the protein expressed in *K. phaffii* (143 kDa). Treatment of the heterologous protein with PNGase F led a band of about 125 kDa, which implied that at least 13% of the total protein mass was due to N-glycosylation, while treatment with α(1 → 2,3,6) mannosidase resulted in a mere 6 kDa (4%) decrease of the protein mass (Fig. 1b, lanes 1 and 2 respectively). The sequential treatment with this PNGase followed by mannosidase, or mannosidase first and then PNGase led to reductions of about 21 kDa (15%) and 15 kDa (11%) in the protein mass, respectively (Fig. 1b, lanes 4 and 5). Regardless of the accessibility that these deglycosylases may have to their cutting targets in GAM1p, all these data pointed to glycosylation constitute at least 15% of the total protein molecular mass.

### Production of isomaltooligosaccharides from maltose

**Fig. 2:**
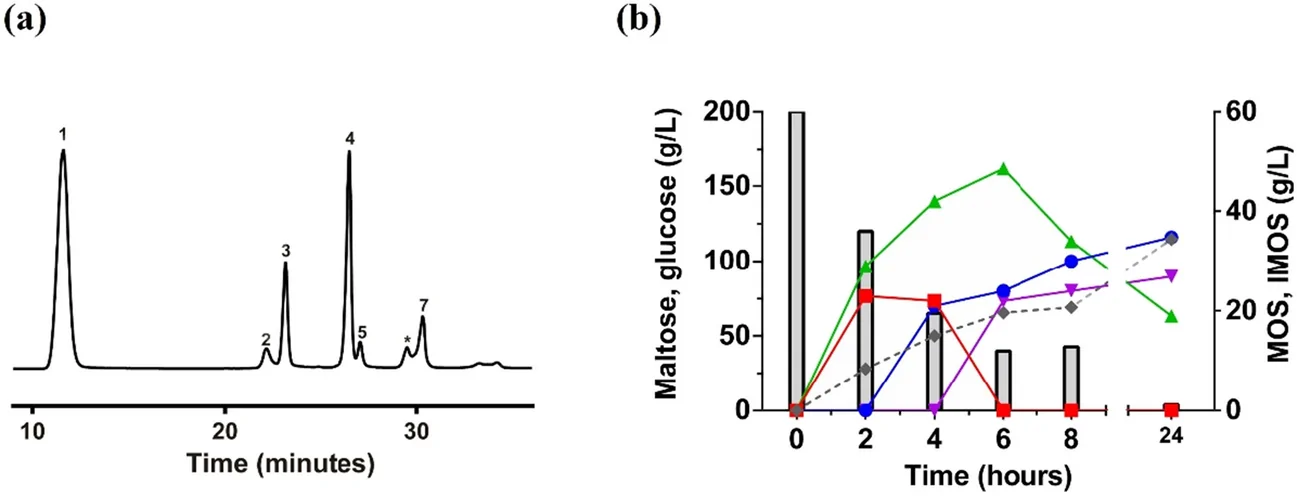
Analysis of the transglycosylation reaction using 200 g/L maltose catalysed by GAM1p. **a** HPLC representative chromatogram of the mixture obtained after 4 h reaction. Peaks correspondence: (1) glucose, (2) maltose, (3) isomaltose, (4) maltotriose, (5) isomaltotriose, (asterisk) no identified, and (7) panose. **b** Evolution of the maltooligosaccharides (MOS) and isomaltooligosaccharides (IMOS) detected throughout the 24 h reaction. Concentrations of maltose (column bars), isomaltose (blue circles) maltotriose (red squares), panose (green triangles), isomaltotriose (purple inverted triangles), and glucose (grey rhombus), obtained at the indicated reaction times, are presented. All sugars were identified and quantified using commercial standards

The transferase activity of GAM1p was first evaluated in reactions containing maltose as the only substrate. The HPLC-ELSD analysis of the mixtures showed chromatogram profiles with numerous peaks (Fig. 2a) corresponding to maltooligosaccharides (MOS)—such as maltotriose—and IMOS, containing α(1 → 6) glycosidic bonds, such as isomaltose, isomaltotriose, and panose. Indeed, the trisaccharides panose and maltotriose were detected from the beginning of the reaction (Fig. 2b), both after linking a glucose unit to maltose by α(1 → 6) or α(1 → 4) glycosidic bonds, respectively. Panose was the major product synthesised, reaching 48.5 g/L after 6 h of reaction and was gradually hydrolysed after reducing the initial concentration of maltose by about 80%.

The synthesis of isomaltose started after the maltose concentration dropped under 100 g/L and increased substantially when panose hydrolysis began. The maximum production of total IMOS (81.3 g/L) was obtained after 24 h of reaction, of which isomaltose (34.8 g/L), isomaltotriose (26.9 g/L), and panose (19.6 g/L) were the main products. At this point, 98% of maltose was hydrolysed.

### Glucose concentration modulates the GAM1p transferase activity

**Fig. 3:**
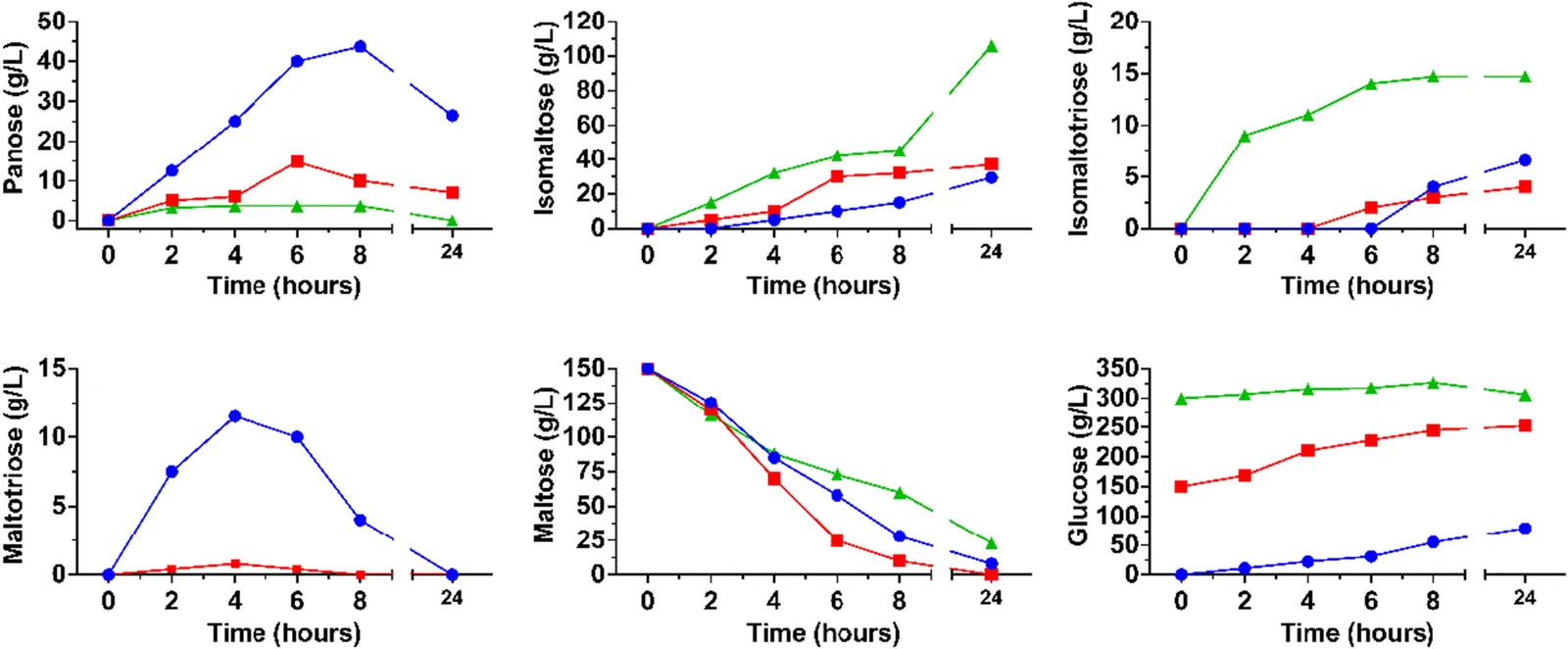
Transglucosylation reactions supplemented with glucose catalysed by GAM1p. Progress of the main di- and trisaccharides produced, as well as residual glucose and maltose evaluated in reactions including GAM1p (25 U/mL) and maltose 150 g/L (control reaction; blue circles); maltose and glucose both 150 g/L (red squares); and maltose 150 g/L and glucose 300 g/L (green triangles). All sugars were identified and quantified using commercial standards

Glucose is undoubtedly a product of the maltose hydrolysis mediated by GAM1p, but it must also act as an acceptor molecule in the production of isomaltose. To confirm this statement, the transferase activity of this protein was analysed in reactions based on maltose (150 g/L) supplemented with glucose and the products obtained were quantified as in the previous section (Fig. 3). The addition of glucose acted drastically on the production levels of all the sugars detected in the reaction. Thus, the maximum concentration of panose dropped from 43.7 g/L to 14.7 and 3.7 g/L when supplementing with 150 and 300 g/L of glucose, respectively. The synthesis of maltotriose was almost completely blocked since only 0.8 g/L of this trisaccharide was produced even at the lowest concentration of glucose used, not being detected at the highest one. Although isomaltose can be produced by degradation of panose, especially at long reaction times, the production of this disaccharide was clearly improved, especially when using 300 g/L of glucose, since an isomaltose concentration of 106 g/L, approximately 3.6 times more than the control (29.3 g/L), was achieved (Fig. 3), accelerating the timeframe for its synthesis. Because the concentration of isomaltose increased at earlier reaction times, the production of isomaltotriose was also improved from the beginning of the reaction, reaching 14.7 g/L of the trisaccharide versus 6.6 g/L of the control reaction without added glucose.

### GAM1 produces heterooligosaccharides

In this work, in addition to exploring the ability of GAM1p to use glucose as transglucosylation acceptor, the saccharides fructose, galactose, glucosamine, xylose, sucrose, cellobiose, and lactose were also assessed. Among all, only sucrose and xylose produced new peaks in the chromatograms of the reactions by HPLC-ELSD.

**Fig. 4:**
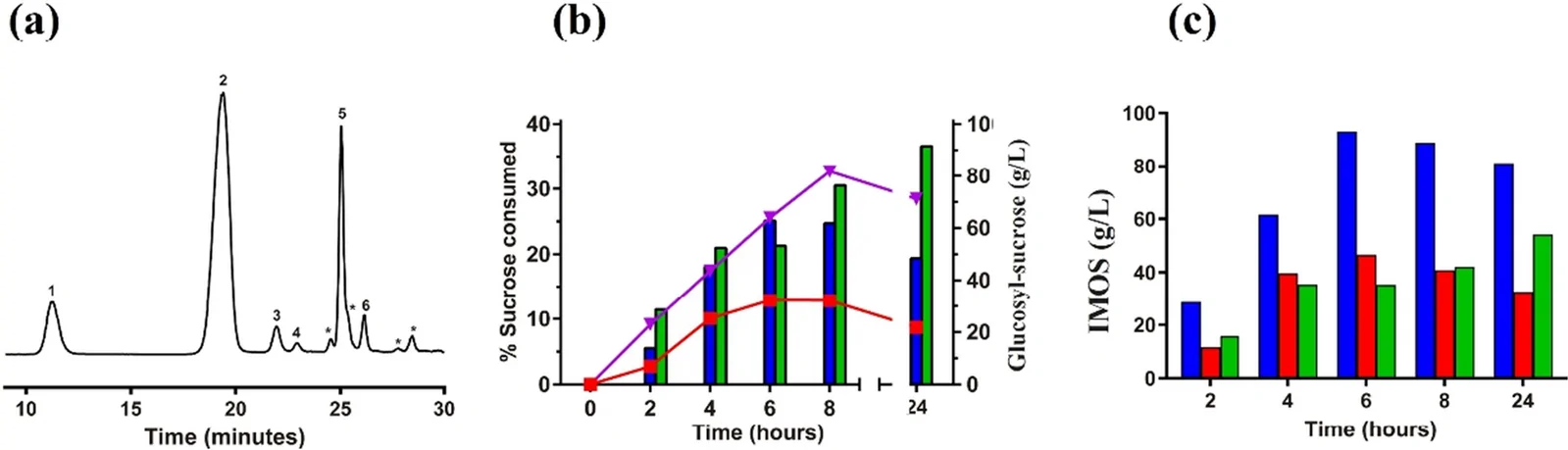
Transglycosylation reaction using sucrose as acceptor. All reactions were performed using GAM1p 25 U/mL and 200 g/L maltose.** a** Representative chromatogram of reaction including maltose 200 g/L and 300 g/L sucrose after 8 h. Peak assignation: (1) glucose; (2) sucrose; (3) maltose; (4) isomaltose; (5) glucosyl-sucrose (identified as theanderose); (6) panose; (asterisk) unidentified compounds. **b** Production of glucosyl-sucrose (theanderose) related to sucrose consumed (%) when using 150 (blue bars) or 300 g/L sucrose (green bars) is indicated with inverted purples triangles and red squares, respectively. **c** IMOS (isomaltose, isomaltotriose, and panose) production in reactions including 0 (blue bars; control), 150 (red bars), or 300 g/L (green bars) sucrose. When possible, sugars were identified and quantified using available commercial standards

The glycosylation of sucrose led to the synthesis of a new major product (Fig. 4a) that was produced from the beginning of the reactions and began to be hydrolysed after 24 h (Fig. 4b). This was purified by a semi-preparative HPLC column and analysed by mass spectrometry electrospray ionisation (MS–ESI), detecting a majority signal of about 527 m/z (M + Na^+^, Fig. S2), which corresponded to the predicted atomic mass of 504 Da for the trisaccharide glucosyl-sucrose. Furthermore, this new product was analysed by NMR and identified as α-D-glucopyranosyl-(1 → 6)-α-D-glucopyranosyl-(1 → 2)-β-D-fructofuranose (theanderose; Fig. S3). Maximum production of theanderose reached 32 and 81.7 g/L by using 150 and 300 g/L sucrose (representing 21.5% and 27.2% of yield), respectively (Fig. 4b), so the production of the heterooligosaccharide increased by 2.5 times by doubling the amount of sucrose in the reaction. The addition of sucrose also altered the synthesis of IMOS, reducing its production by at least 50% after 6 h of reaction (Fig. 4c), and curiously this was partially recovered after 24 h when using the highest concentration of the disaccharide.

**Fig. 5:**
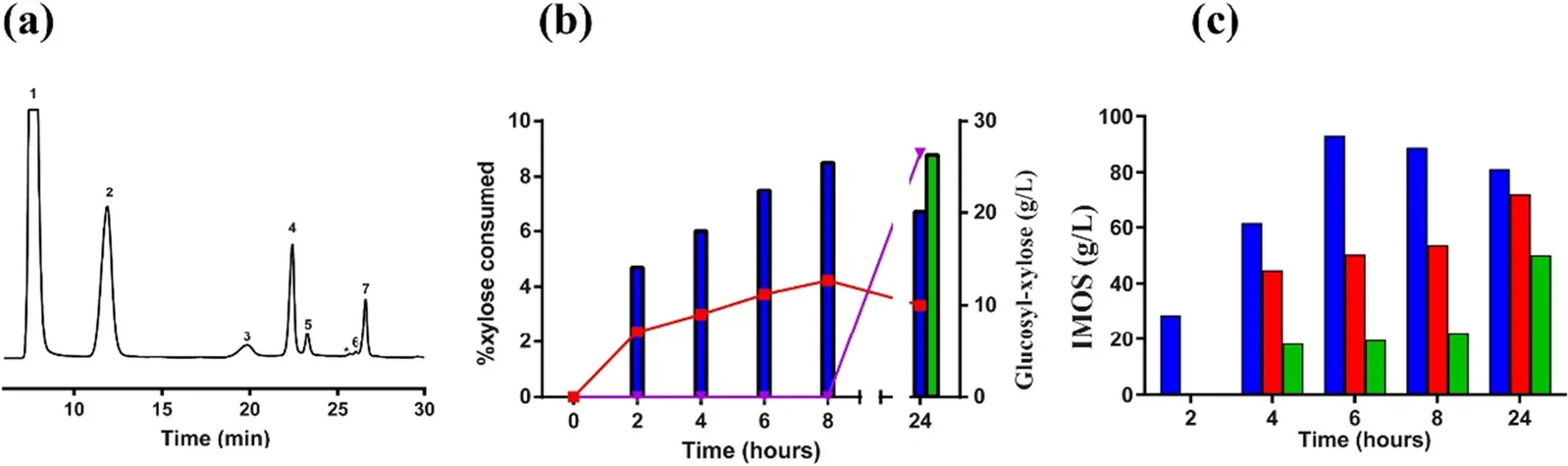
Transglycosylation reaction using xylose as acceptor. All reactions were performed with GAM1p 25 U/mL and maltose 200 g/L. **a** Chromatogram of transglycosylation products of the reaction including xylose 300 g/L after 24 h. Peak assignation: (1) xylose; (2) glucose; (3) glucosyl-xylose (4) maltose; (5) isomaltose; (6) maltotriose; (7) panose; (asterisk) unidentified compound. **b** Glucosyl-xylose production related to the percentage of xylose consumed when using xylose 150 (blue bars) and 300 g/L (green bars) in red squares and inverted purple triangles, respectively. **c** IMOS (isomaltose, isomaltotriose, and panose) production in reactions including 0 (blue bars; control), 150 (red bars), and 300 g/L (green bars) xylose. When possible, sugars were identified and quantified using available commercial standards

Only one major glycosylation product was detected in the reaction mixture when using xylose in the reactions (Fig. 5a). The maximum amount produced of this new compound was 12.7 g/L after 8 h and 26.5 g/L after 24 h of reaction using 150 g/L and 300 g/L of xylose, respectively (Fig. 5b). The new compound was purified by semi-preparative HPLC and then analysed by MS–ESI as before. A major signal of 335 m/z (M + Na^+^) was detected, which corresponded to the predicted atomic mass of 312 Da for the disaccharide glucosyl-xylose (Fig. S4). This glycosylation product turned out to be α-D-glucopyranosyl-(1 → 4)-α-D-xylopyranose as deduced by NMR, detecting the alpha and beta anomers of xylopyranose (Fig. S5). In addition, another minor product was also formed, which was identified as the non-reducing disaccharide α-D-glucopyranosyl-(1 → 1)-β-D-xylopyranose.

The presence of xylose in the reaction also caused a reduction in the IMOS production (Fig. 5c). In fact, no IMOS were detected after 2 h reaction; isomaltose was only slightly detected after 4 h using the lowest concentration of xylose and after 24 h at the highest one, with no isomaltotriose detected at any reaction time for this last case (data not shown). Furthermore, in both xylose conditions, the synthesis of IMOS was partially reversed after 24 h, occurring simultaneously the lowest concentration of xylose with the decrease in the glucosyl-xylose production.

### Glycosylation of polyphenols

The capacity of GAM1p to glycosylate phenolic compounds was analysed using epigallocatechin gallate (EGCG), hydroquinone, hydroxytyrosol, and piceid. Since the solubility of piceid in water is limited, a cosolvent (DMSO) was added to favour the transglucosylation reactions. The GAM1p hydrolytic activity was analysed including 20% DMSO in the reaction mixture, remaining stable after 4 h and reduced by 20% and 30% after 7 h and 24 h incubation, respectively (Fig. S6).

**Fig. 6:**
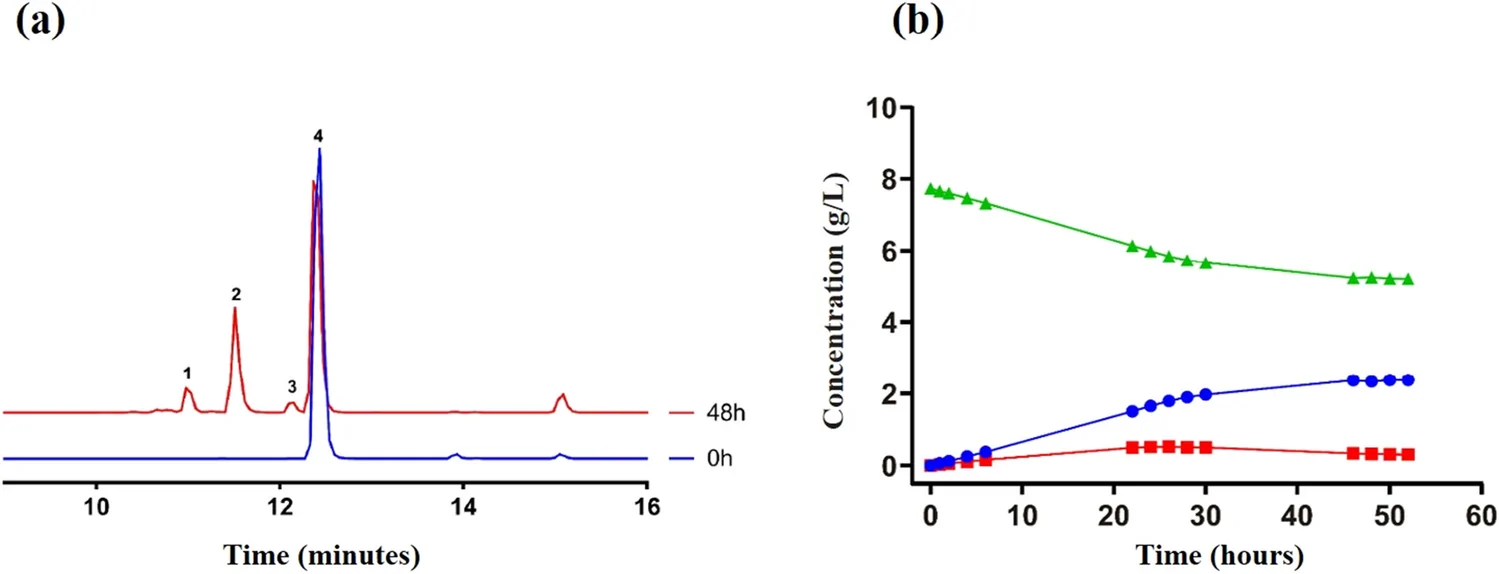
Analysis of the piceid transglycosylation. Reaction included GAM1p 50 U/mL, piceid 20 mM, maltose 300 g/L, and DMSO 20% (v/v) in sodium acetate 100 mM pH 4.5. **a** HPLC-PAD chromatograms obtained at the indicated reaction times. Peak assignation: (1) piceid 6,6′-diglucoside; (2) piceid 6-monoglucoside; (3) piceid 4-monoglucoside; (4) piceid. **b** The reaction was incubated at 37 °C and the aliquots at the indicated times were analysed by HPLC–PDA. Concentration of piceid (green triangles), piceid monoglucoside (blue circles), and piceid diglucoside (red squares) at the indicated reaction times are shown

Reactions were monitored by HPLC-PAD during 52 h and new chromatographic signals were only detected when using piceid (also called polydatin) (Fig. 6a), a natural monoglycosylated derivative of resveratrol. In the chromatograms of the reactions that contained piceid, three new signals were detected, peaks 1, 2, and 3 (Fig. 6a). The MS–ESI analysis of peaks 1 and 2 (previously purified by semi-preparative HPLC) showed values of m/z of 737 and 575, respectively, which corresponded to the M + Na^+^ ions of piceid diglucoside (714 Da) and piceid monoglucoside (552 Da) (Fig. S7 and S8). Peak 3 corresponded to a minor monoglucosylated product (contaminated with piceid in the purification process, Fig. S9). Therefore, the major glycosylation product detected in the reaction was piceid monoglucoside, which reached 3.4 g/L after 46 h (Fig. 6b). This molecule generated by GAM1p was glycosylated again, forming piceid diglucoside, about 1 g/L at the same reaction time.

Analysis by NMR of the products formed by the piceid glycosylation showed that, in both the piceid diglucoside and monoglucoside, the new glucose units were linked by α(1 → 6) bonds to the preceding glucose unit, with ratios of the cis/trans configurations for the stilbene double bond of 1:1.2 and 2:5, respectively (Fig. S10 and Fig. S11). In the minor product (peak 3 in Fig. 6a), the piceid monoglucoside showed the glucose moiety connected by α(1 → 4) linkage. Also, the presence of geometric *cis/trans* isomers was detected for this molecular entity, although they could not be quantified due to the overlapping of the *trans* isomer signals with those of the major product (Fig. S12).

## Discussion

The protein sequence of the α-glucosidase GAM1p from the yeast *S. occidentalis* showed the characteristic barrel structure (β/α)8 of the family GH31, and in general a great structural similarity with the α-glucosidase of *Beta vulgaris* and the N-terminal subunit of the human intestinal maltase-glucoamylase and sucrase-isomaltase. The conserved sequences Wi/nDMNEa/p/v and HWt/l/gGDNa/tA were also located in the vicinity of the two catalytic aspartates, both the nucleophile (D472) and the proton donor (D640) responsible for the acid–base reaction of this enzyme (Fig. S13). GAM1p was expressed extracellularly in *K. phaffii* using a general strategy that was already used to successfully produce different GHs from yeast and fungi, including the α-glucosidase XdGlu and the β-fructofuranosidase XdINV from *Xanthophyllomyces dendrorhous* (Gimeno-Pérez et al. 2015; Gutiérrez-Alonso et al. 2016), the β-fructofuranosidase RdINV from *Rhodotorula dairenensis* (Gimeno-Pérez et al. 2021; Jiménez-Ortega et al. 2022), and the chitinases Chit42 and Chit33 from *Trichoderma harzianum* (Kidibule et al. 2018, 2020). In all these cases, and as it occurs with GAM1p, no extracellular proteins were detected in the culture filtrates of control yeasts transformed with the empty vector pIB4. The maximum amount of the protein GAM1p produced in this work reached 70 mg/L at 70–78 h of the yeast culture and was apparently purified after a simple concentration step of the extracellular medium (Fig. 1b). Data that represent an increase of about 10 times in the production of GAM1p was previously obtained in *P. pastoris* (Sato et al. 2005), where only about 0.2 U/mL of α-glucosidase at 29.5 U/mg was obtained, which represented a production of GAM1p lower than 7 mg/L of yeast culture. These same authors showed that this protein was glycosylated, estimating a molecular weight for the protein of 130 kDa and 126 kDa after PNGase F treatment. In our case, the protein treated with this deglycosylase also showed a size very similar to this (125 kDa), and it was reduced even a little more (122 kDa) after the sequential treatment with mannosidase. However, despite these small differences in the degree of glycosylation, the enzyme produced in *K. phaffii* does not appear to have significantly altered its kinetic properties when using maltose as a substrate. Our kinetic analysis showed a catalytic efficiency (*k*_cat_/*K*_m_) of 1615 ± 30 s^*−*1^ mM^*−*1^ (data not shown), the same as previously referred using maltose as substrate (Sato et al. 2005; Song et al. 2013).

Microbial α-glucosidases show very frequently glycosyltransferase activity when using saturating substrate conditions and produce IMOS as well as MOS (mainly maltotriose) from maltose (Kato et al. 2002; Wu et al. 2010). The latter a trisaccharide that is efficiently hydrolysed by the intestinal glycosyl hydrolases and therefore lacking prebiotic properties (Lee et al. 2014). IMOS formed by enzymes of the family GH31, as GAM1p, contain at least one α(1 → 6) bond, although some of these enzymes also form sugars including α(1 → 1) or α(1 → 3) linkages such as kojibiose or nigerose (Kawano et al. 2020; Saburi et al. 2015; Wu et al. 2010).

**Fig. 7:**
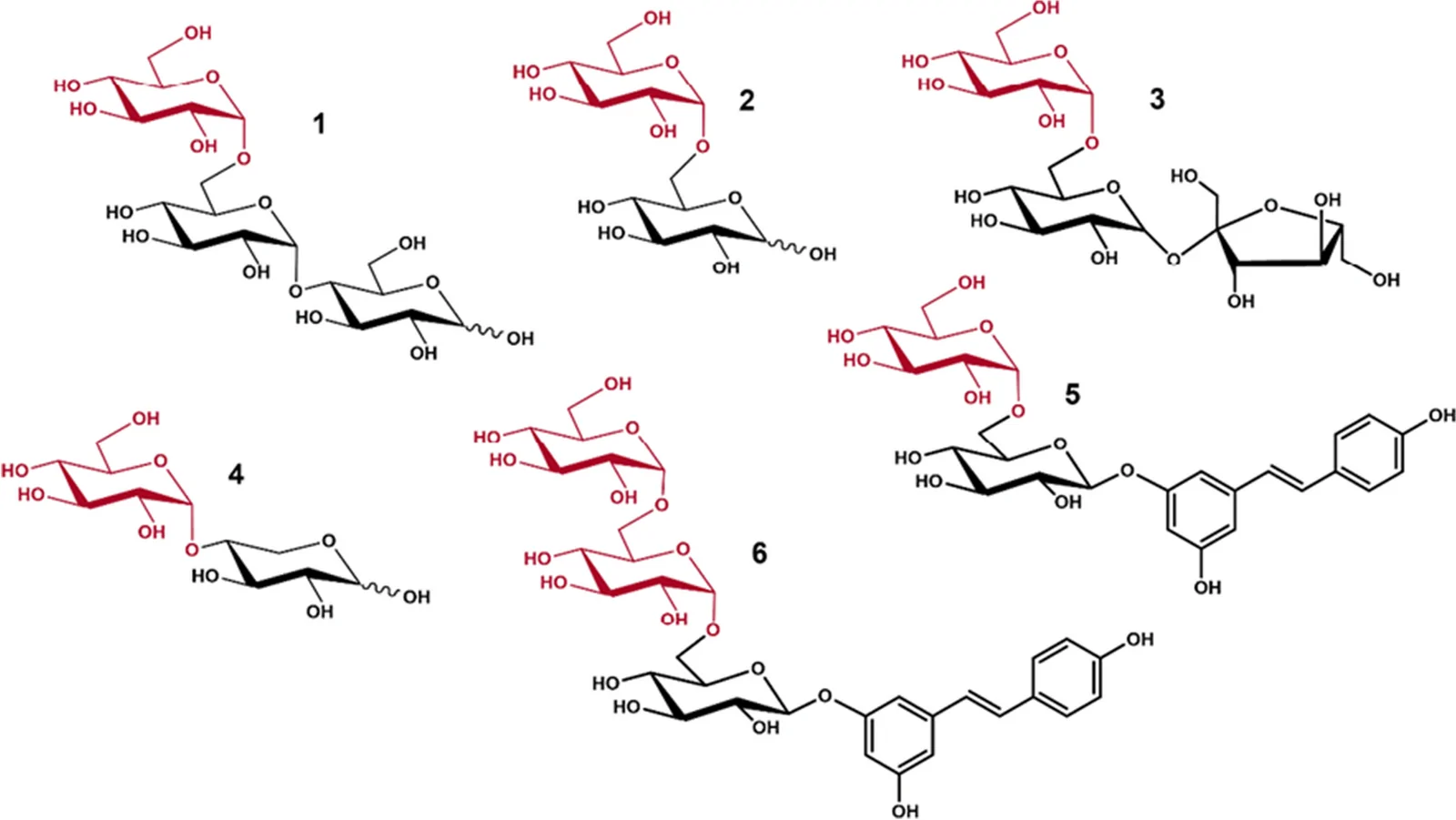
Structure of the main transglycosylation products obtained with GAM1p. (1) Panose. (2) Isomaltose. (3) Theanderose. (4) α-D-Glc-(1 → 4)-Xyl. (5) Piceid 6-monoglucoside. (6) Piceid 6,6′-diglucoside. The transferred α-glucosyl moieties are indicated in red colour

The transglucosylation activity of GAM1p was studied in detail in this work, using different sugars and polyphenols as acceptors. Figure 7 shows the main transglycosylation products obtained. First, when using maltose as only substrate, GAM1p mainly produces panose (Glc-α(1 → 6)-Glc-α(1 → 4)-Glc) and isomaltose (Glc-α(1 → 6)-Glc), the first by transfer of a glucose unit to maltose. Panose starts undergoing hydrolysis as the maltose concentration diminishes (Fig. 2). However, synthesis of isomaltose a priori occurs both by secondary hydrolysis of panose and by the glucosylation of free glucose through an α(1 → 6) bond. In fact, addition of glucose promoted the formation of this type of linkage since it notably increased the production of isomaltose and isomaltotriose and practically eliminated that of maltotriose (Fig. 3). In this context, the α-glucosidase of *Aspergillus niger* also produced panose and isomaltose as main maltose transglycosylation products (Casa-Villegas et al. 2017). However, and although reaction with this last enzyme was followed only during 5 h, isomaltose synthesis was not increased as notably (1.6 times; 17 to 27 g/L) as with GAM1p (4.3 times; 9 to 39 g/L after 5 h, and 3.6 times, 29 to 106 g/L after 24 h). The α-glucosidase of *Aspergillus neoniger* also produces panose and isomaltose as main glycosylation products with yields of about 0.21 and 0.07 g/g maltose, respectively (Kumar et al. 2020), versus about 0.24 and 0.17 g/g maltose obtained by GAM1p, emphasising the high potential of GAM1p to produce isomaltose. In animals, mixtures of IMOS are partially digested and used by the intestinal microbiota, favouring the proliferation/metabolism of bifidobacteria and lactobacilli (Hu et al. 2020; Kaneko et al. 1994; Ketabi et al. 2011). The fact that GAM1p mainly forms sugars with α(1 → 6) bonds, at long reaction times, especially isomaltose and isomaltotriose, and that in addition, the production of these sugars increases as the glucose concentration rises, boosts the industrial potential of the protein to produce IMOS that could be used for human consumption or in animal feed.

Some glycosyl hydrolases also form heterooligosaccharides by using their natural substrate together with alternative carbohydrate acceptors (Chen et al. 2019; Nimpiboon et al. 2011). In the present work, GAM1p glucosylated sucrose and xylose but not fructose, galactose, and glucosamine. Cellobiose was also apparently glycosylated (not hydrolysed) but unfortunately the compounds formed seemed to coelute with maltose, so it could not be quantified (data not shown) or structurally resolved to demonstrate that GAM1p could glycosylate acceptors with β-glycosidic bonds.

When using sucrose, the main acceptor product was theanderose (Fig. 7), a non-cariogenic sugar of prebiotic potential (Ruíz-Aceituno et al. 2017) that is found mainly in honey, being its extraction from this natural source not very profitable (up to 0.3 g per 100 g of honey; Ruiz-Matute et al. 2010). The family GH13 includes several α-glucosidases showing sucrose splitting activity, and those from *Bacillus* sp. and *Metshnikowia* spp. also synthesise theanderose by using sucrose as a substrate, forming about 60 g/L (Garcia-Gonzalez et al. 2020; Inohara-Ochiai et al. 2000) compared with the 82 g/L produced by GAM1p. Site-directed mutagenesis was also applied to the *Bacillus* sp. enzyme to increase 1.7 times (to about 104 g/L) the formation of theanderose (Okada et al. 2002). As far as we know, the largest amount of theanderose produced (approx. 130 g/L) was achieved with the levansucrase from *Bacillus subtilis*, using sucrose as donor of fructose units and isomaltose—an expensive sugar—as acceptor (Ruíz-Aceituno et al. 2017).

GAM1p also glucosylated xylose, an aldopentose, yielding α-D-glucopyranosyl-(1 → 4)-α-D-xylopyranose and a very small amount of α-D-glucopyranosyl-(1 → 1)-α-D-xylopyranose (Figs. 5 and 7). The synthesis of the first compound was already described a long time ago by using a maltose phosphorylase from *Neisseria meningitidis* (Putman et al. 1955). However, the chemical characterisation of this disaccharide was not described, and structure was only proposed. The related compound in beta configuration Glc-β(1 → 4)-Xyl was obtained by using *Cellvibrio gilvus* cells containing cellobiose phosphorylase (Kumagai, et al. 2010). On the other hand, production of the minor compound obtained with GAM1p Glc-α(1 → 1)-α-Xyl was previously described employing a trehalase from *Trichoderma reesei* (Kasumi et al. 1986) or a chemical strategy (Nishizawa et al. 1994).

Concerning the tested phenolic compounds, GAM1p was only able to glycosylate piceid (polydatin), but no EGCG, hydroquinone, and hydroxytyrosol. This is probably due to the presence in the piceid of a glucose moiety β-linked to the resveratrol unit that serves as an anchoring point (Figs. 6 and 7). Although the productions of the glycosylated piceid derivatives were quite low compared with that of the IMOS and heterooligosaccharides (about 3.4 g/L and 1/g/L for the monoglycosylated and diglycosylated derivatives, respectively), the conversion yield was about 56% (Fig. 6b). To our concern, the α(1 → 6) mono- and diglucosides obtained in this work are novel compounds. In this context, glucosyl-α(1 → 4)-piceid was described using an amylosucrase from *Alteromonas macleodii* (Park et al. 2012). Glucosides of piceid were also synthesised by transglycosylation using the cyclodextrin glucanotransferase (CGTase) from *Bacillus macerans*, with maltodextrin as glucosyl donor (Mathew et al. 2012); however, the synthesised compounds (mono- and diglucoside) were only characterised by MS but not by NMR, and the specificity of CGTase usually gives rise to α(1 → 4) linkages (Martin et al. 2004). In fact, the formation of an α(1 → 4) glucosylated derivative of piceid using a CGTase was later described (Shimoda et al. 2015). Piceid is interesting from a biotechnological point of view because of its notable antioxidant activity, which is related with numerous potential bioactivities through the modulation of pivotal signalling pathways involved in inflammation, oxidative stress, and apoptosis (Karami et al. 2022), making it a potential candidate for the treatment of cancer (Farooq et al. 2023) and some mental diseases such as Alzheimer’s (Tang 2021; Xiao et al. 2015). Glycosylation of piceid increases its aqueous solubility, which may favour its bioavailability. Unfortunately, the structural determinants of proteins that can use alternative glycosylation acceptors, and especially polyphenolic compounds, are not yet well characterised, nor is the structure of the α-glucosidase from *S. occidentalis*. Objective we are already working on. Determining the relationship that exists between the structure–function of proteins, such as in this case the GH31 α-glucosidases, is key to knowing their biological role and their possible biotechnological improvement and applications.

## Supplementary Information

Below is the link to the electronic supplementary material.

Supplementary file1 (PDF 1619 KB)

## Data Availability

All data generated or analysed during this study are included in this manuscript.
