# Supplementary material for: Insights into the transglucosylation activity of α-glucosidase from Schwanniomyces occidentalis
Source: Appl Microbiol Biotechnol. 2024 Aug 17;108:443. doi: 10.1007/s00253-024-13262-8 (PMC11330417; doi:10.1007/s00253-024-13262-8)
Supplement: Supplementary file 1 [file 253_2024_13262_MOESM1_ESM.pdf]

Supplementary Materials

Insights into the transglucosylation activity of α-glucosidase from *Schwanniomyces occidentalis*

Zoran Merdzo[1], Egle Narmontaite[1], Jose L Gonzalez-Alfonso[2], Ana Poveda[3], Jesus Jimenez-Barbero[3], Francisco J. Plou[2], María Fernández-Lobato[1*]

Figure S1	SDS-PAGE (10%) analysis of the GAMp expression in *Pichia pastoris*.
Figure S2	MS-ESI analysis of the major product of sucrose transglycosylation.
Figure S3	NMR analysis of the major product obtained by glucosylation of sucrose (Theanderose)
Figure S4	MS-ESI analysis of the major product of xylose transglycosylation
Figure S5	NMR analysis of the major product obtained by glucosylation of xylose.
Figure S6	Stability of GAM1p in DMSO.
Figure S7	MS-ESI analysis of the diglycosylated piceid obtained by transglycosylation.
Figure S8	MS-ESI analysis of the monoglycosylated piceid obtained by transglycosylation.
Figure S9	MS-ESI analysis of the transglycosylation product of resveratrol.
Figure S10	Aromatic region of the ^{1}H-NMR spectra of Piceid, Glc-Piceid and di-Glc Piceid samples.
Figure S11	ROESY and HSQC spectrum of piceid monoglucoside and diglucoside.
Figure S12	Assignment of the minor glucosylated product of piceid (contaminated with piceid).
Figure S13	GAM1p Structural Alignment.

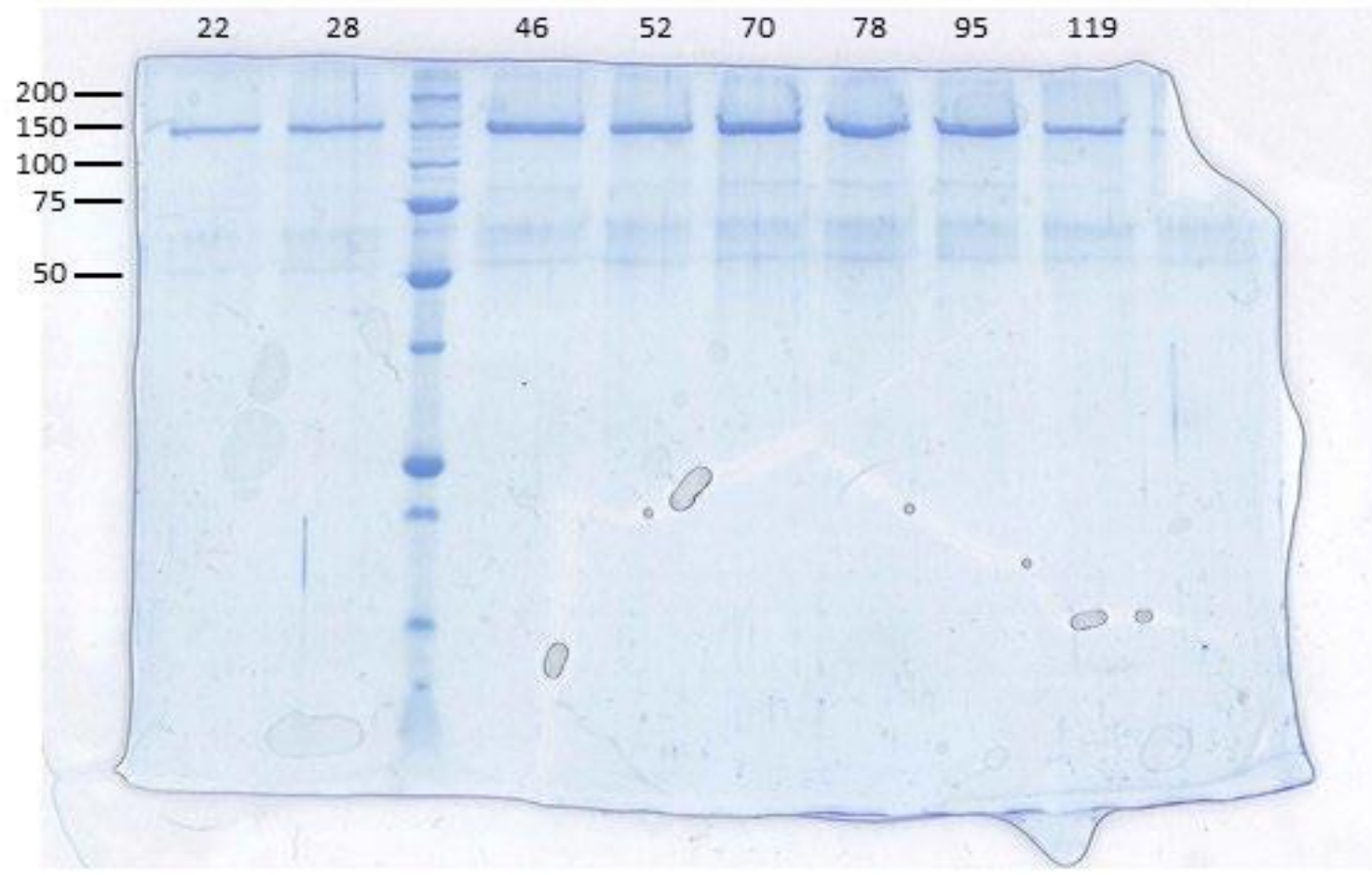

Figure S1. SDS-PAGE (10%) analysis of the GAM expression in *Pichia pastoris*. The majority band of almost 145 kDa corresponds to the heterologous protein, that was not detected in yeast transformants including the empty pIB4 vector. Extracellular medium of the transformant (10 µL) including the GAM1-pIB4 construction and cultured in BMM were loaded at the indicated times (in h). Numbers on the left indicate positions of molecular mass standards (lane between 28-46 h) in kDa.

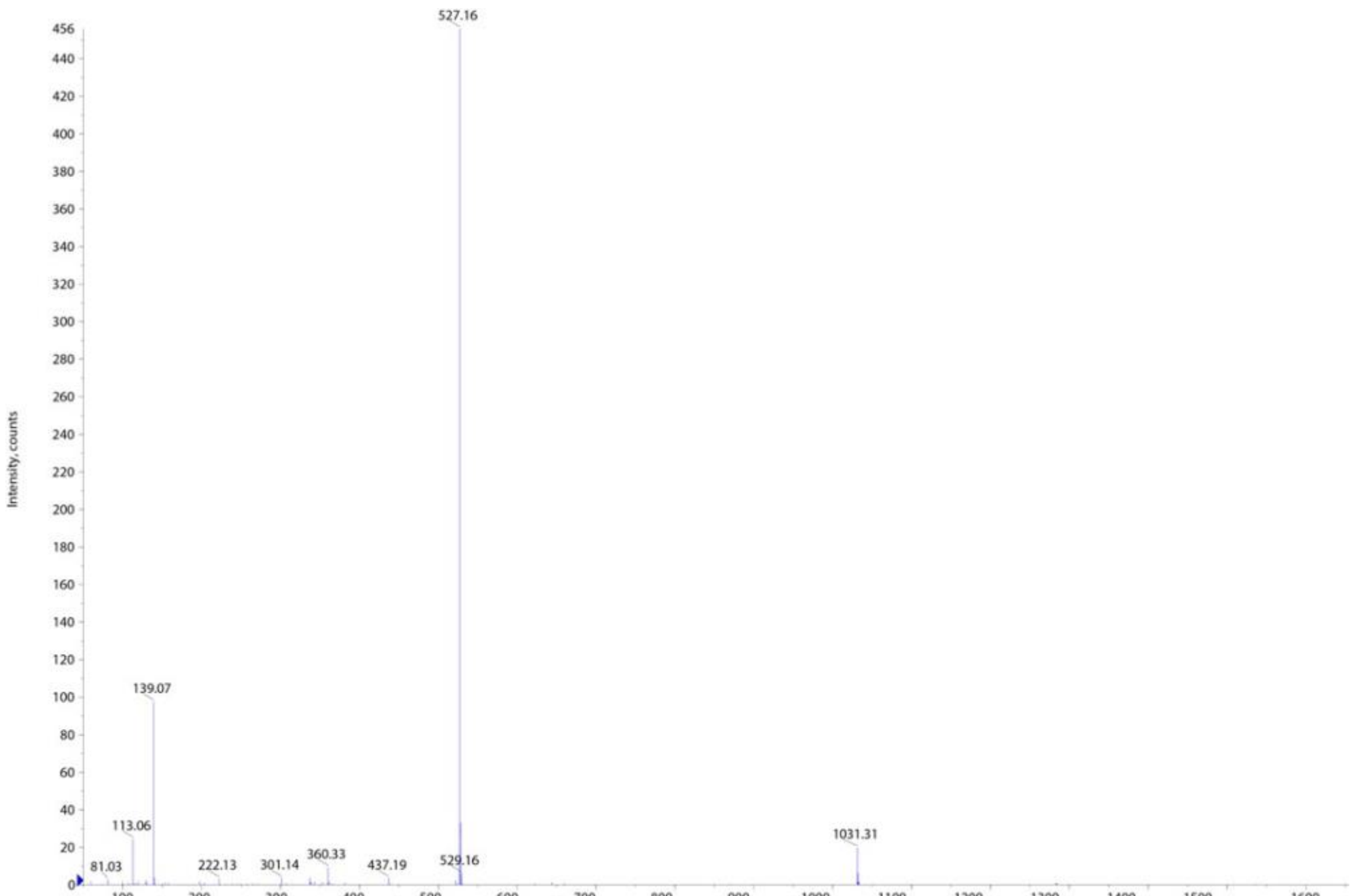

Figure S2. MS-ESI analysis of the major product of sucrose transglycosylation. The majority sugar produced was purified with a semipreparative column using HPLC. The majority signal of about 527 m/z corresponded to the predicted atomic mass of a trisaccharide, 504 Da + sodium adducts (due to the use of NaI as ionizing phase in the test).

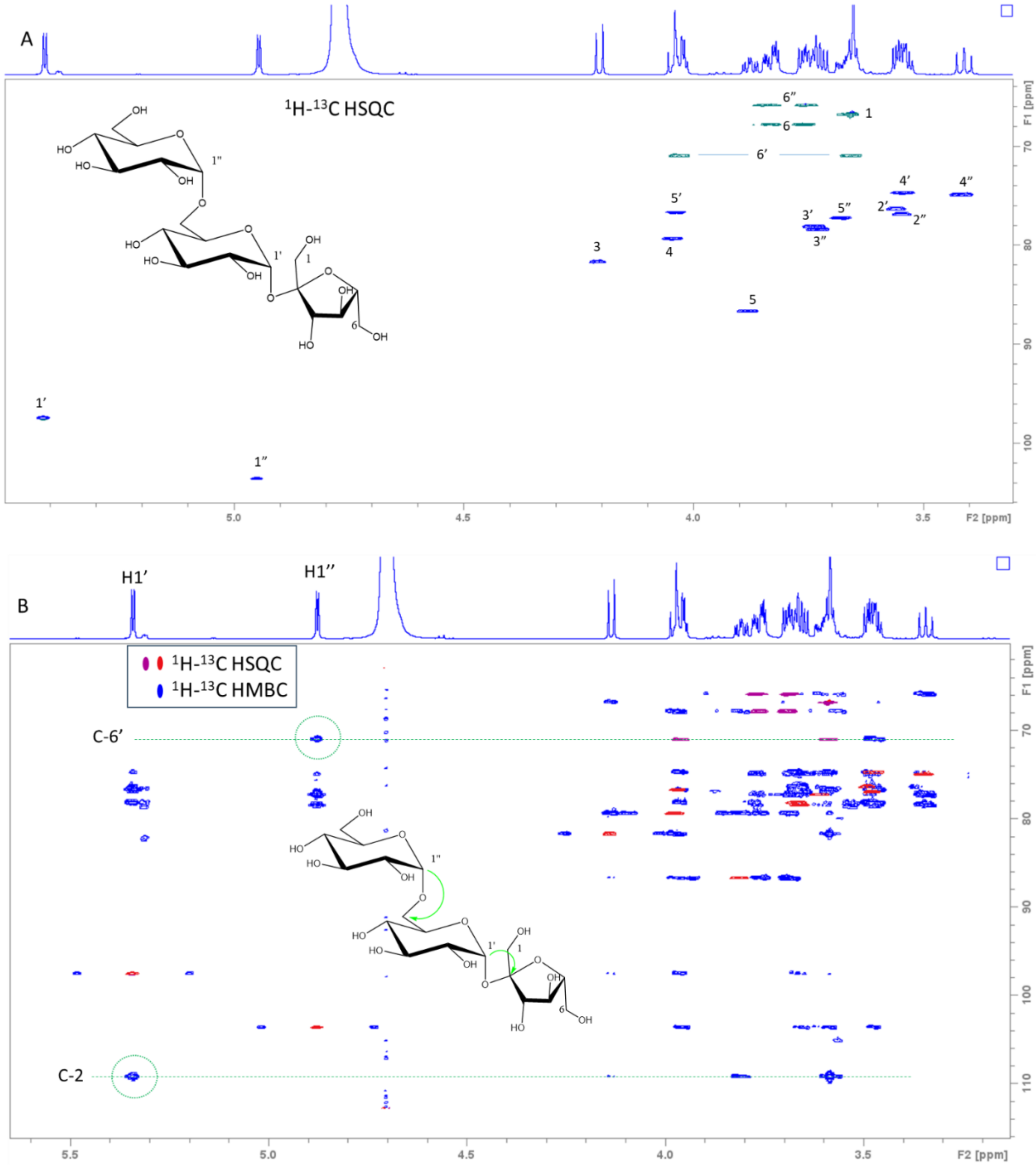

Figure S3. NMR analysis of the major product obtained by glucosylation of sucrose. (A) ^{1}H-^{13}C HSQC spectrum with the complete assignments of the NMR signals. (B) Superimposition of ^{1}H-^{13}C HMBC (blue) and ^{1}H-^{13}C HSQC (red, violet) spectra, highlighting the key glycosylation correlations: the H1' anomeric proton from original Glc ring correlates with the quaternary carbon C-2 of fructose ring (as in sucrose) and anomeric proton H1'' from the incorporated Glc ring correlates with the carbon C-6' of the glucose ring originally belonging to the sucrose, giving rise to theanderose trisaccharide.

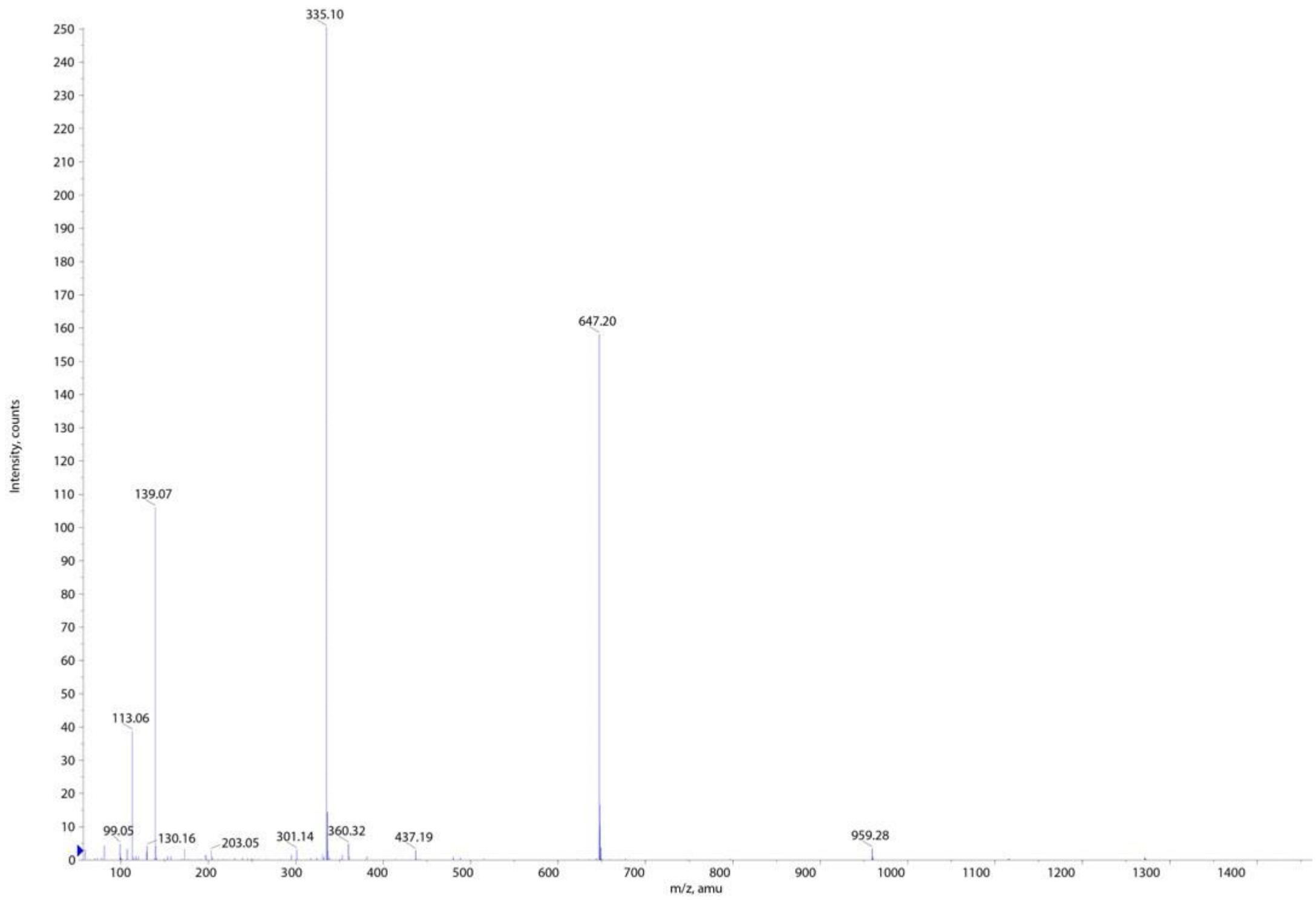

Figure S4. MS-ESI analysis of the major product of xylose transglycosylation. Analysis of the purified major sugar with a semipreparative column using HPLC. The majority signal of about 335 m/z corresponded to the predicted atomic mass of the disaccharide glucosyl-xylose, 312 Da + sodium adducts (due to the use of NaI as ionizing phase in the test).

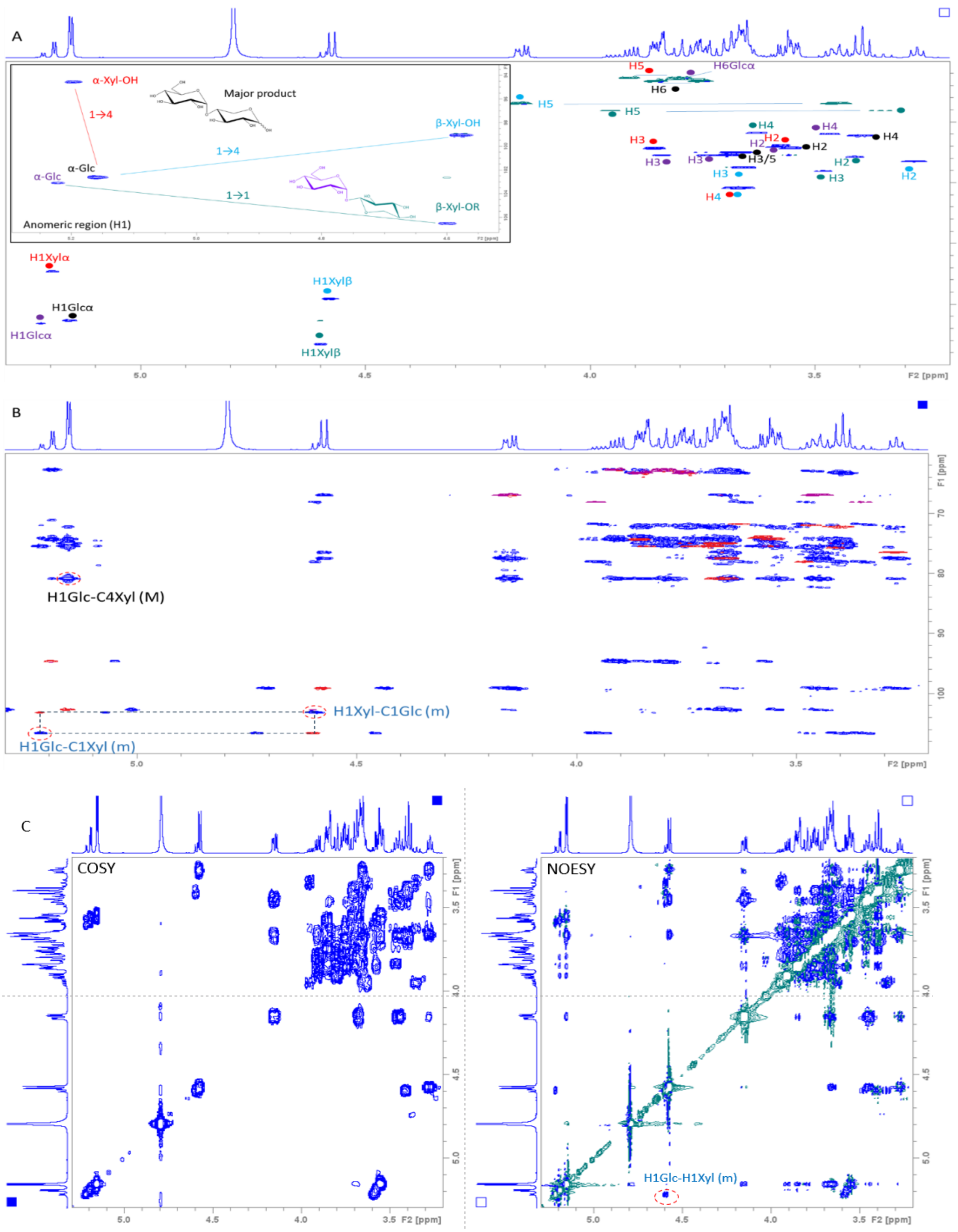

Figure S5. NMR analysis of the major product obtained by glucosylation of xylose. (A) ^{1}H-^{13}C HSQC spectrum with complete assignments of the NMR signals. (B) Superimposition of ^{1}H-^{13}C HMBC (blue) and ^{1}H-^{13}C HSQC (red, violet) spectra, with the key glycosylation correlations highlighted: The anomeric proton H1 from the major Glc moiety correlates with carbon C-4 of the xylose ring (both α and β residues) giving rise to α-D-glucopyranosyl-(1-4)-α-D-xylopyranose. For the minor product, the anomeric proton H1

from the Glc ring correlates with the carbon C-1 of the Xylose ring with β configuration, giving rise to α-D-glucopyranosyl-(1-1)-β-D-xylopyranose.(C) The analysis of the COSY (left) and NOESY (right) spectra allowed assessing the H1Glc-H1Xyl NOE found for the minor product.

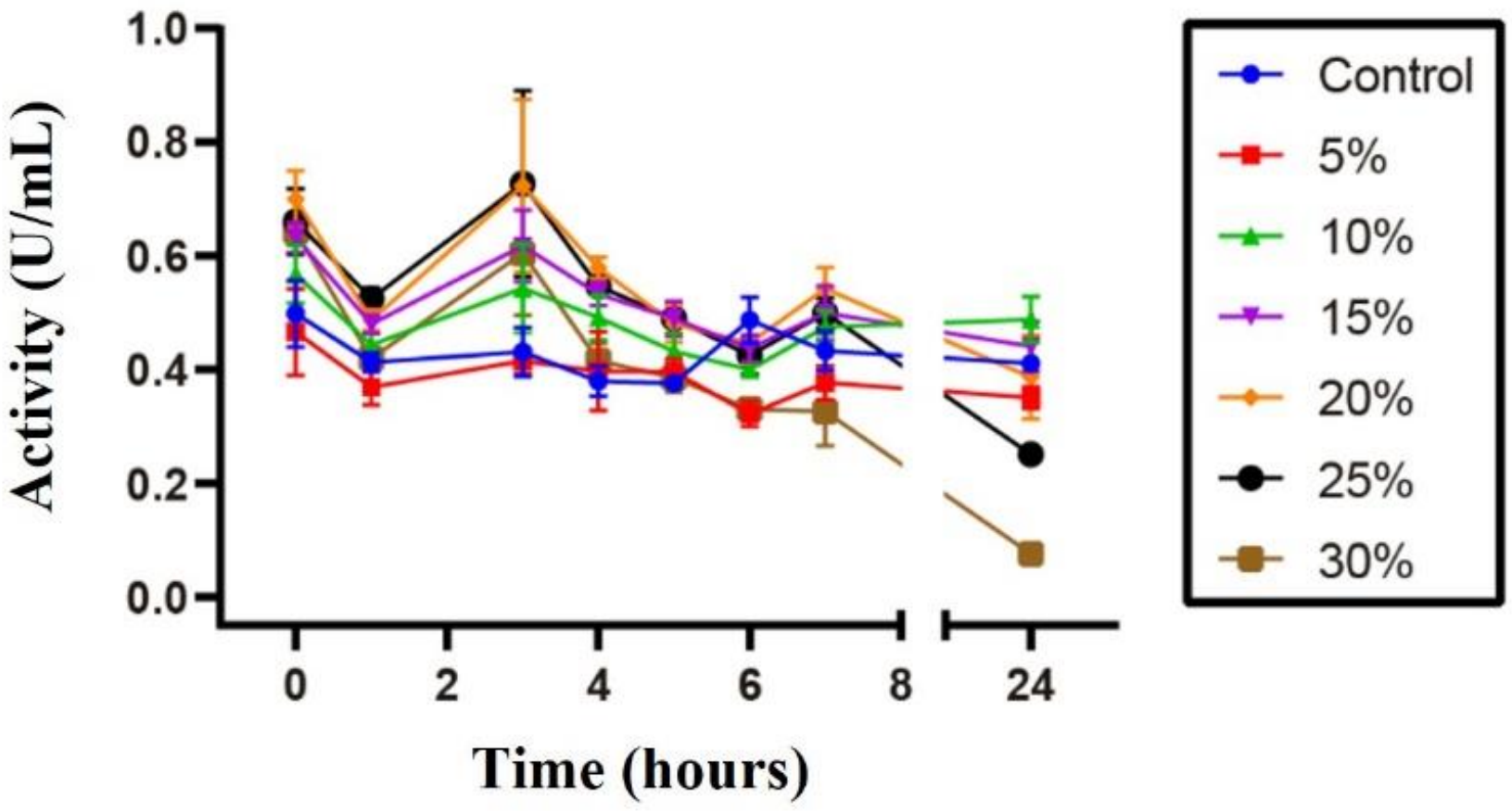

Figure S6. Stability of GAM1 in Dimethyl sulfoxide (DMSO). GAM1- was maintained at 37ºC in the referred concentrations of DMSO (v/v) buffered with 50 mM sodium acetate pH 4.5 after which the activity was analyzed using 2 mM maltose. The control was incubated with the buffer alone.

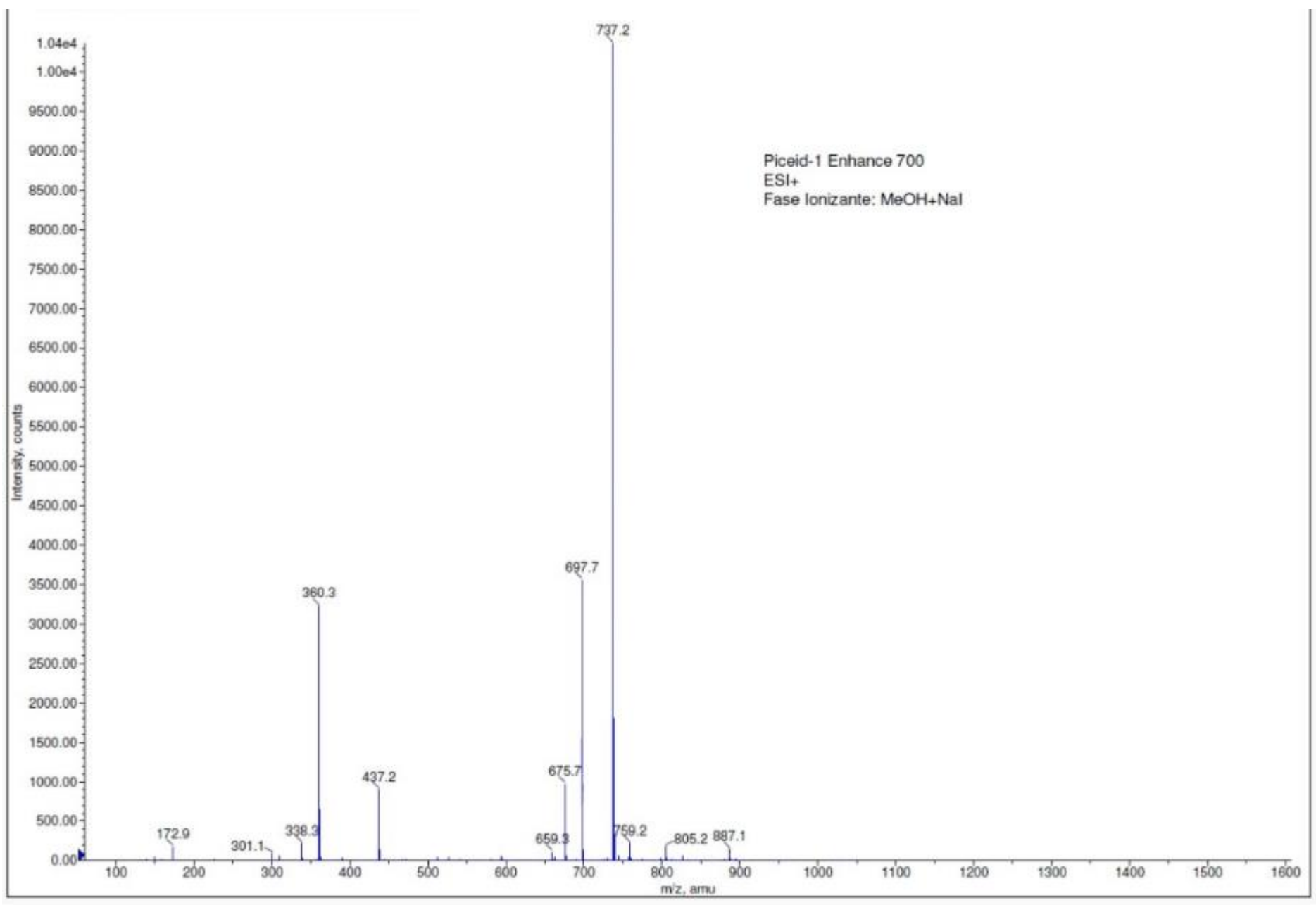

Figure S7. MS-ESI analysis of the diglycosylated piceid obtained by transglycosylation. Product purified by semipreparative column using HPLC from peak 1 of the chromatogram showed in Figure 6a. The majority signal of about 737 m/z corresponded to the predicted atomic mass of the diglycosylated piceid, 714 Da + sodium adducts (due to the use of NaI as ionizing phase in the test).

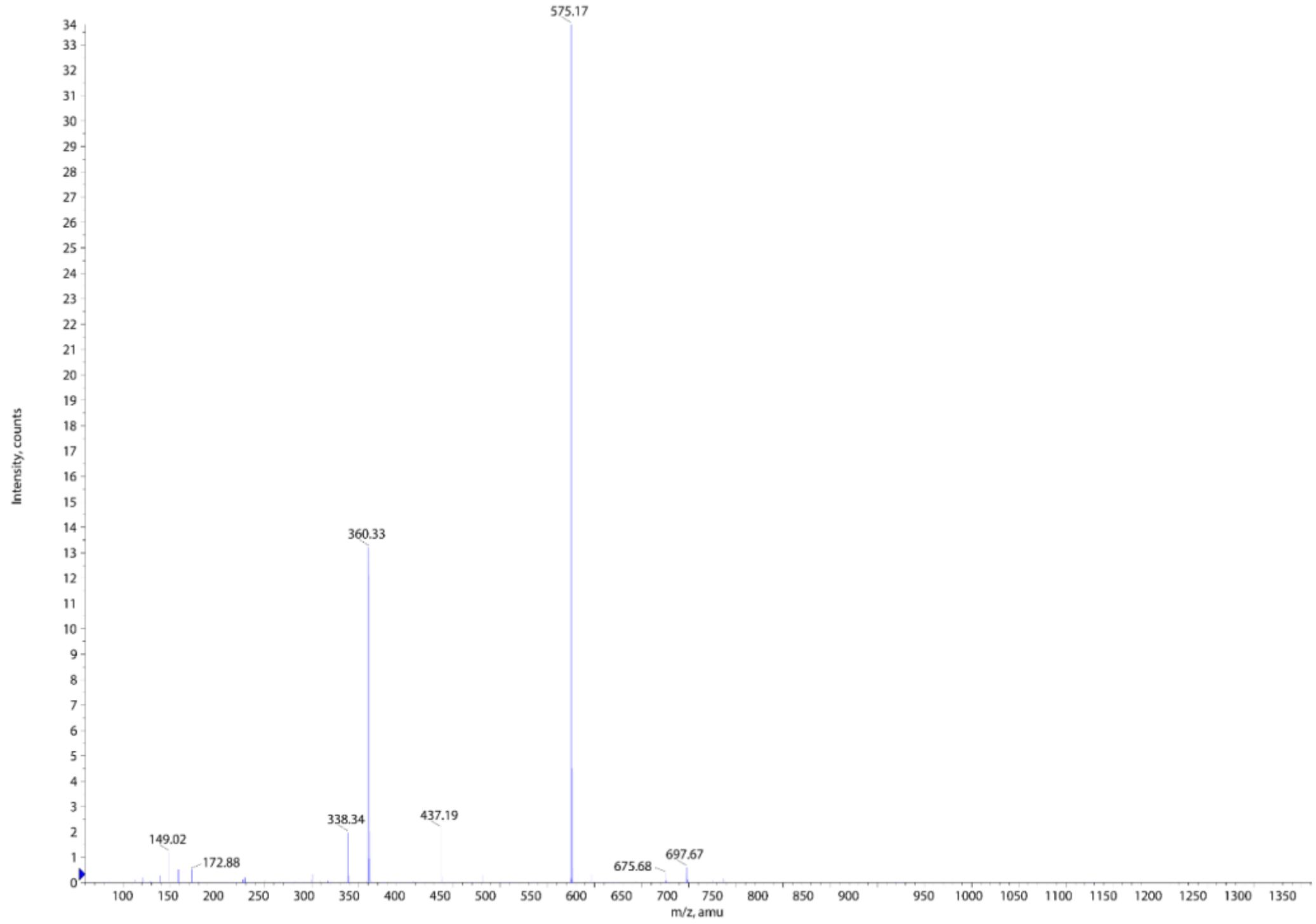

Figure S8. MS-ESI analysis of the monoglycosylated piceid obtained by transglycosylation. Product purified by semipreparative column by HPLC from peak 2 of the chromatogram showed in Figure 6a. The majority signal of about 575 m/z corresponded to the predicted atomic mass of the monoglycosylated piceid, 552 Da + sodium adducts (due to the use of NaI as ionizing phase in the test).

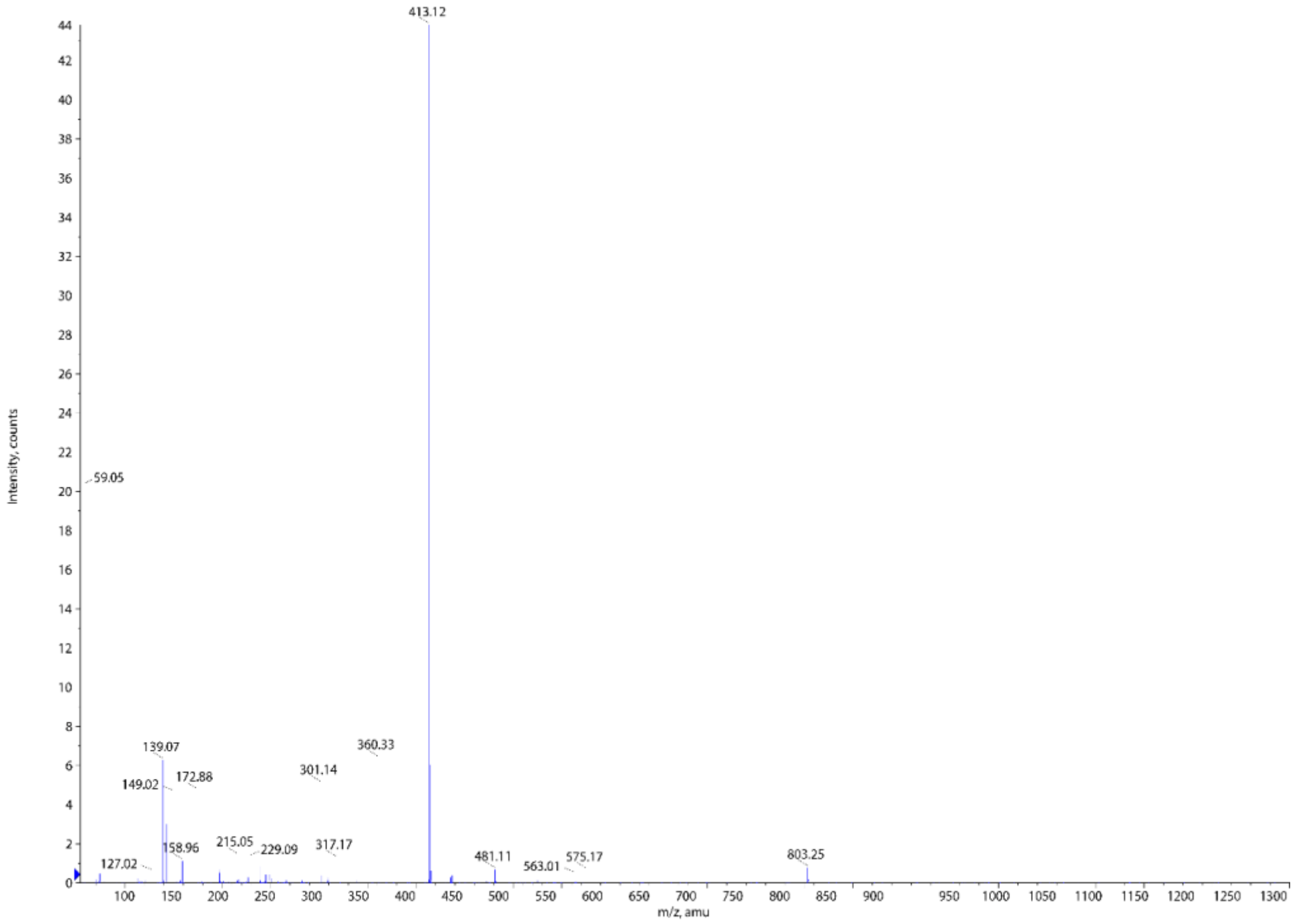

Figure S9. MS-ESI analysis of the transglycosylation product of resveratrol. Product purified by se mipreparative column using HPLC from peak 3 of the chromatogram showed in Figure 6a. The majority signal of about 413 m/z corresponded to the predicted atomic mass of the monoglycosylated resveratrol, 390 Da + sodium adducts (due to the use of NaI as ionizing phase in the test).

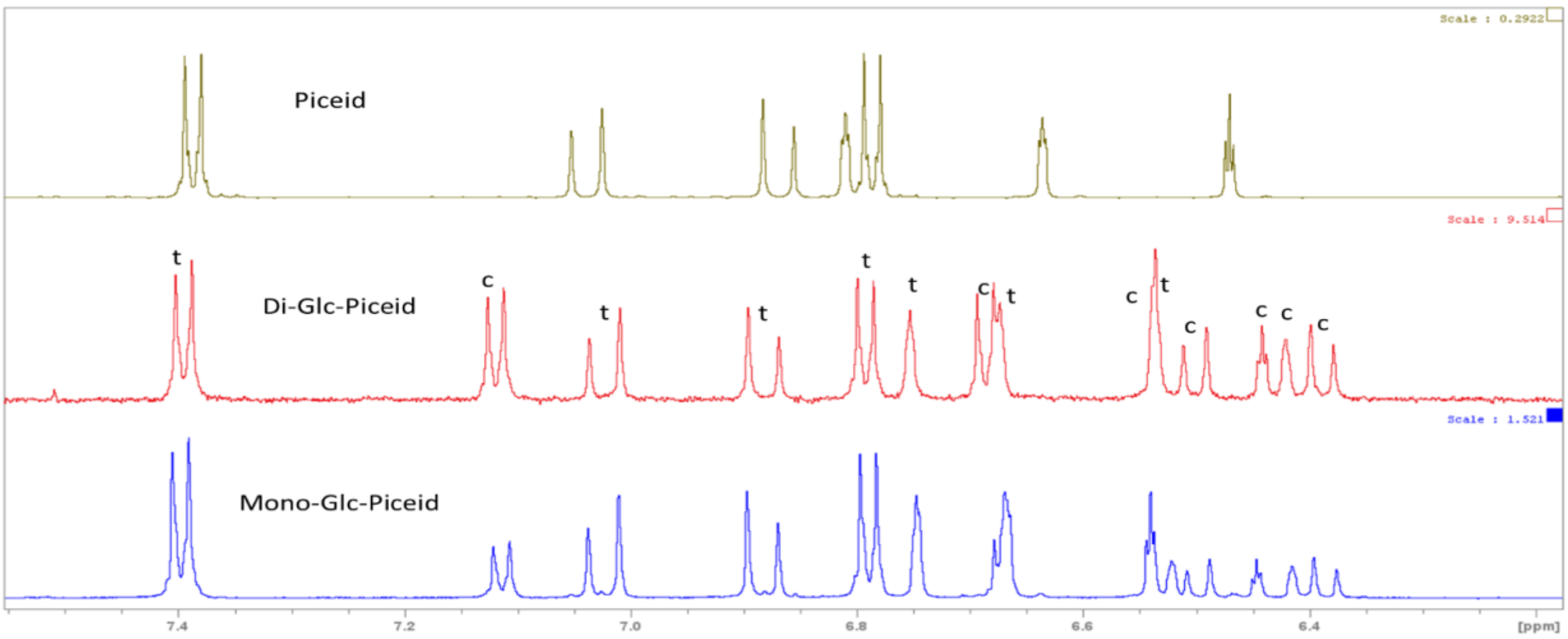

Figure S10. Aromatic region of the ^{1}H-NMR spectra of Piceid, Glc-Piceid and di-Glc Piceid samples. For the glucosylated samples, both the *cis (c)* and *trans (t)* piceid moieties at the double bond are present.

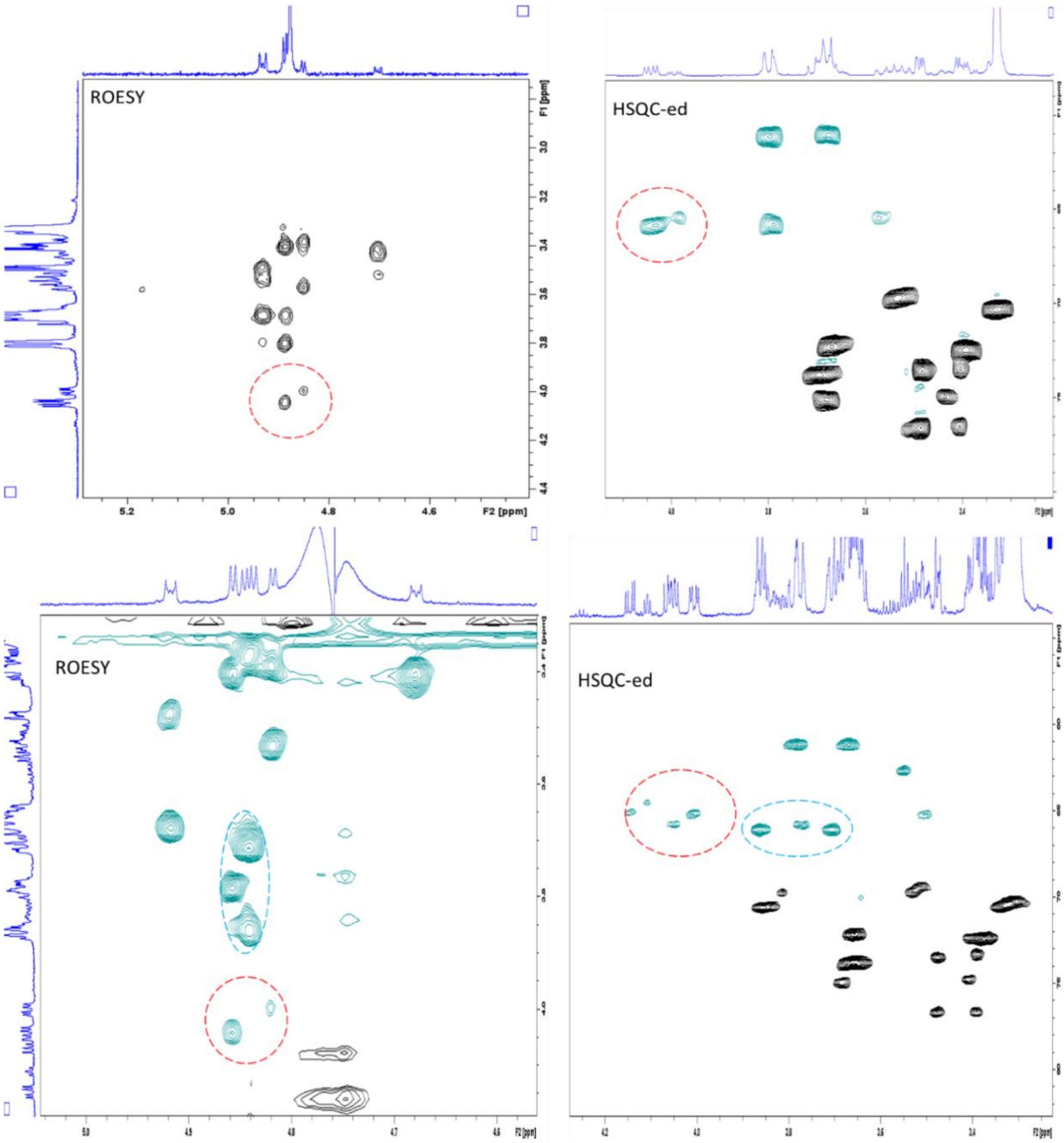

Figure S11. Partial ROESY and HSQC spectrum of piceid monoglucoside (top panels) and diglucoside (bottom panels). The ROESY spectra indicate that the Glc is linked by an α-(1-6) glycosidic bond and the HSQC indicate that the methylene groups appear at ^{13}C chemical shifts of 65 ppm, as corresponds to CH_2-O- glycosylated groups.

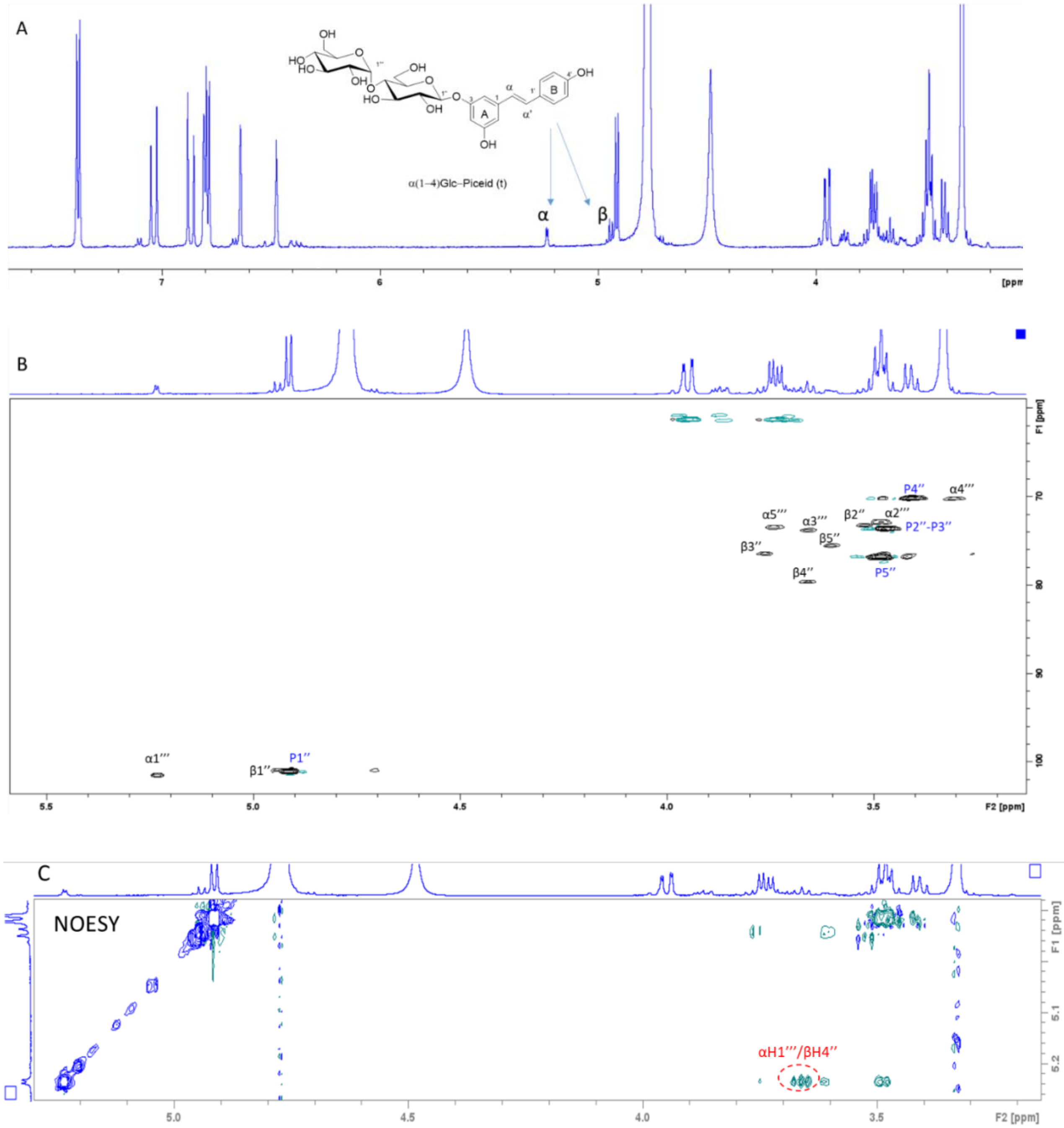

Figure S12. Assignment of the minor glucosylated product of piceid (contaminated with piceid): (A) ^{1}H-NMR spectrum, (B) Partial HSQC spectrum of the sugars region. The signals of a new glucose unit linked to the piceid motive through α-(1-4) linkage were identified (black labels). Piceid major product signals are labelled in blue. (C) Section of NOESY spectrum showing the H1'''-H4'' NOE that confirms the 1-4 linkage.

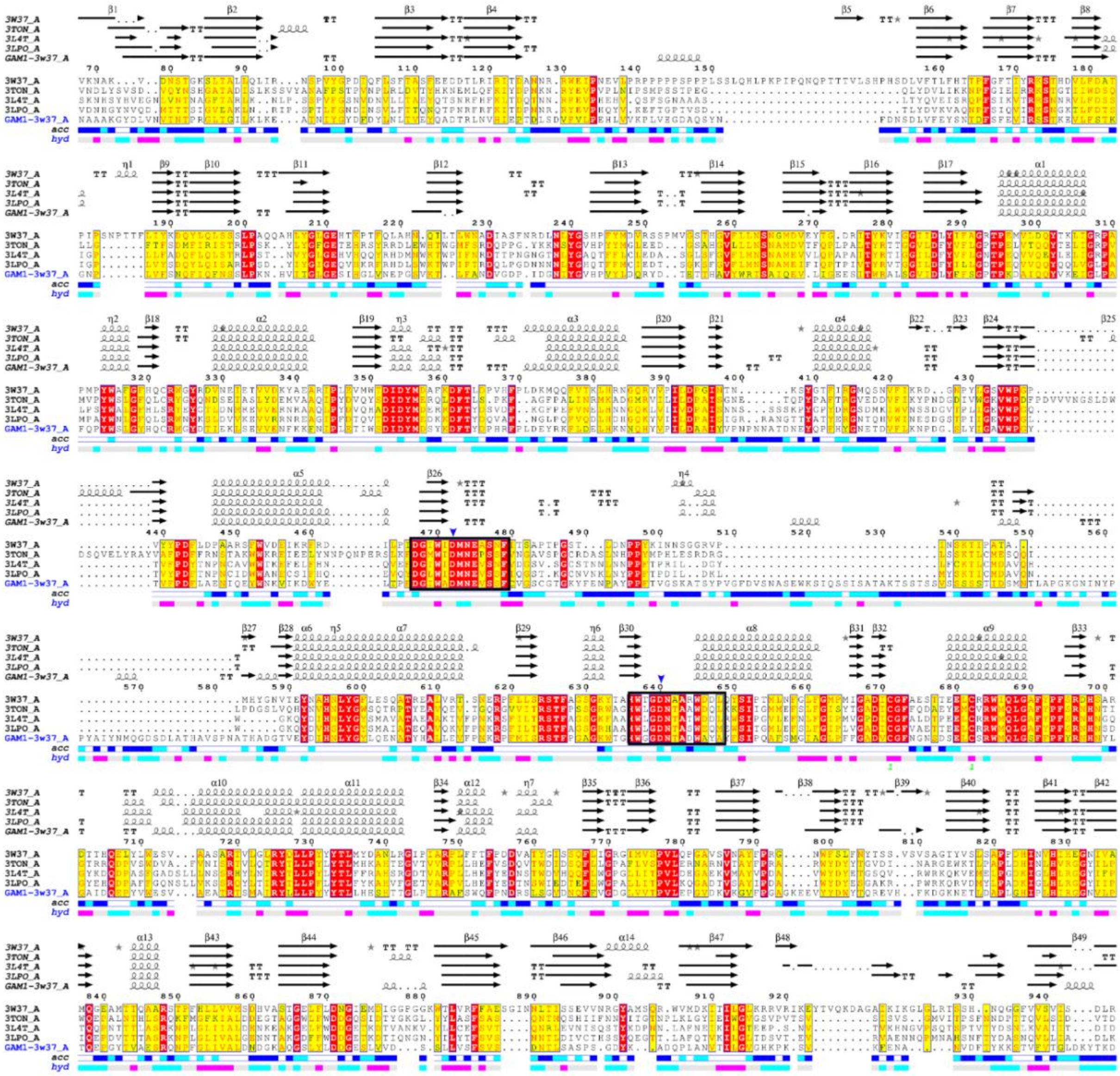

Figure S13. GAM1p Structural Alignment. Result of the alignment of sequences of the GAM1p model created with reference to the α-glucosidase of *Beta vulgaris* against the 47 most homologous sequences obtained by ENDscript 2.0 (only the 4 sequences with the best alignment are shown). The secondary structures are shown at the top (helices with spirals, β-sheets with arrows, and turns with the letters TT). Similar residues are marked in yellow, identical residues in red, and catalytic residues with a blue arrow pointing downward at the top. The conserved motifs of the catalytic region are framed in black. The representative sequences shown are: α-glucosidase of *B. vulgaris* (PDB: 3W37), the C-terminal (PDB: 3TON) and N-terminal domains of human maltase-glucoamylase (PDB: 3L4T) and of human sucrase-isomaltase (PDB: 3LPO), respectively.
